# Predicting enviromically adapted varieties with big data

**DOI:** 10.1186/s13059-025-03914-x

**Published:** 2026-01-07

**Authors:** Abhishek Gogna, Bahareh Kamali, Valentin Wimmer, Renate H. Schmidt, Ehsan Eyshi Rezaei, Wera Maria Eckhoff, Jochen C. Reif, Yusheng Zhao

**Affiliations:** 1 Leibniz Institute of Plant Genetics and Crop Plant Research, Corrensstraße, Gatersleben, 306466 Germany; 2 KWS SAAT SE & Co. KGaA, Grimsehlstraße, 31, Einbeck, 37574 Germany; 3 Institute of Crop Science and Resource Conservation, Katzenburgweg, 9a, Bonn, 53115 Germany; 4 Leibniz Centre for Agricultural Landscape Research, Eberswalder, Straße 84, Müncheberg, 15374 Germany

**Keywords:** Winter wheat, Breeding programs, Genotype performance, Big data, Artificial intelligence, Machine learning, Deep learning, Genotype times environment interactions, Enviromically adapted varieties

## Abstract

**Background:**

Breeding programs prioritize the average performance of a genotype across environments and may overlook promising candidates for specific environments. To address this challenge, we propose a genomic prediction framework to select high-yielding genotypes tailored to individual environments.

**Results:**

We compiled winter wheat grain yield data from 13,285 genotypes—6,766 lines and 6,519 hybrids—evaluated in yield plots at 31 central european sites from 2010 to 2022. With integrated genomic data, we show that only as the size of the training dataset increase, convolutional neural networks benchmark competitive to superior compared with traditional genomic best linear unbiased predictions (GBLUP) in predicting average genotype performance of lines. We then extend our prediction models to account for genotype times environment (G × E) interactions by incorporating information about the growth environment. We observe a 23% improvement in predicting environment-specific performance of new hybrids within a network of test environments with GBLUP based models. To better understand the environmental variables driving G × E interactions, we conduct analyses on a core set of 500 genetically diverse lines. Using machine learning, we successfully identify pivotal environment variables driving the clustering of study environments in central europe and highlight the benefit of modelling G × E interactions in selection of enviromically adapted varieties.

**Conclusions:**

Our results suggest that big data in combination with machine learning and deep learning methods offers new ways to widen the genetic bottleneck often encountered when advancing candidates from early limited-environment to late stage multi-environment evaluations. This promises faster delivery of breeding progress to farmers’ fields.

**Supplementary Information:**

The online version contains supplementary material available at 10.1186/s13059-025-03914-x.

## Background

Global wheat production faces significant challenges, primarily stemming from the rapid diversification of growing environments due to climate change. The latter translates directly into fluctuating local yields of crop varieties, evidenced by a genetic yield gap of approximately 40 percent for winter wheat, even in high-yielding countries such as Germany [1]. To counteract the diffused productivity of elite varieties, it is essential to reconsider candidate selection in breeding programs. Typically, only promising candidates are evaluated in multi-environment yield trials, and a few top performers are then registered for national listing [2]. This approach creates a genetic bottleneck, potentially overlooking candidates best suited for specific target environments.

The selection of genotypes for grain yield averaged across environments is routinely supported by genome-wide predictions [3]. These are based on genotypic effects estimated in extensive training populations, commonly using models such as Genomic best linear unbiased predictions (GBLUP) [4], and are particularly useful for complex quantitative traits like grain yield. However, to predict how a genotype will perform under local on-farm conditions, genotype times environment (G × E) effects must also be considered. G × E interaction is the variable effect of growth environment–shaped by climate, soil, and crop management–on genotype performance and may lead to change in merit of selected candidates [5, 6]. Traditional approaches to account for G × E effects have used variance–covariance matrices in best linear unbiased prediction models. However, these models require estimating matrix components from the data, making them unsuitable for predictions in unobserved environments [7, 8]. To overcome this, some studies have used reaction norm models combining genomic information with environment variables, aggregated either by seasonal periods [9, 10] or specific stages of crop growth as derived from crop growth models (CGMs) [11, 12]. Alternatively, CGMs informed by genomic information have been used to explicitly account for the non-linear nature of G × E interactions in predicting grain yield [13]. Artificial intelligence approaches, including machine learning methods such as random forests [14, 15] and deep learning methods like convolutional neural networks [16] have also been proposed for this purpose. Notably, isolated small to medium sized public datasets, often with limited genetic diversity, have dominated the G × E interactions research so far and potential of systematic data fusion, especially via public–private partnerships, enabling Big Data has not yet been explored in wheat, a major food grain crop.

The concept of Big Data in plant breeding domain introduces new approaches to managing information by applying FAIR principles [17]—findable, accessible, interoperable, and reusable—for integrating diverse data types beyond genotypic and phenotypic information [18]. Since the data generated in the domain is often highly heterogeneous, the shift from ‘Data’ to ‘Big Data’ is primarily process-driven, focusing on methods to standardize, connect, and analyze complex datasets [19]. While product-related components—relating to the volume, velocity, and variety of data itself—can often be adapted from other fields, process-oriented challenges like trait standardization, multi-environment data integration, etc. require solutions largely developed from within the domain.

As a first step toward this goal, we augmented an existing wheat Big Data resource [20] with additional phenotypic and genomic information. Line and hybrid genotypes in our data were characterized with around 10,000 genotypic markers and evaluated in field trials across Central Europe, organized into seven experimental series. We characterized the growth environments using 297 environment variables describing seasonal weather conditions i.e. daily temperature, wind speed, humidity, and others. We then developed model frameworks starting with the prediction of average genotype performance and later extending to prediction of environment-specific performance (called henceforth enviromically adapted). For the latter, we also parameterized genotype growth at specific trial sites with additional information, including soil and crop management data, using the crop growth model MONICA [21].

Based on Big Data, we studied environment clusters in Central Europe driven by G × E interactions and identified key environment variables that explain G × E patterns within a growing season. We then predicted grain yield performance of a reference set and assessed fluctuations in genotype rankings in relation to changes in target environments. Our results suggest that, compared to candidates selected for overall performance, substantial yield increases can be achieved by growing enviromically adapted genotypes in the target environments. With an average genetic yield gain of 0.32 Q ha^−1^ year^−1^, which has been estimated for winter wheat in Germany [22], projected yield boosts equaled up to 12 years of realized breeding progress. Therefore, we propose that leveraging Big Data for modeling G × E interactions and incorporating the selection of enviromically adapted genotypes into the breeder’s toolbox can facilitate the development of climate-smart varieties, thereby helping to close the genetic yield gap.

## Results

### Data fusion revealed ample genetic and environmental diversity

**Fig. 1:**
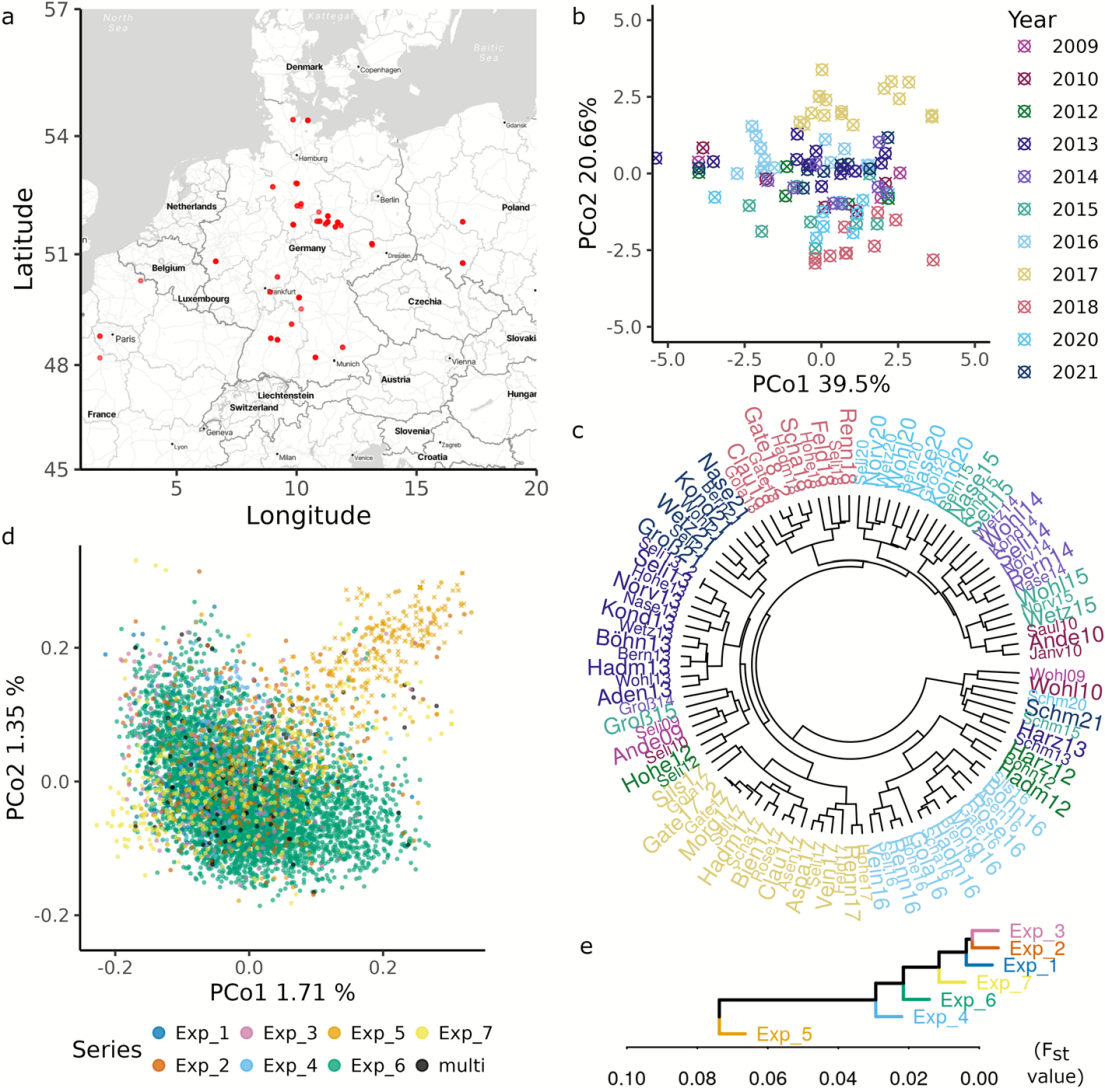
Phenotypic data were collected from field trials conducted in Central Europe, with trial sites indicated on the map in subfigure (**a**). Environmental variables (EV) were derived from climate data and environment diversity space was visualized using a principal coordinates (PCo) plot based on EV pairwise Euclidean distances in subfigure (**b**). Major environmental clusters, identified through hierarchical clustering on EVs, are shown in subfigure (**c**), with environments in (**b**) and (**c**) colored by year as indicated in the legend of (**b**). The genetic diversity of the lines evaluated in the trials is represented by a PCo plot of the Rogers’ distance matrix, calculated using integrated genotypic data, in subfigure (**d**). In this plot, the points are color-coded based on the experimental series to which each line belongs (Exp_1 through Exp_7 or multiple series). Additionally, males from Exp_5 are highlighted with green crosses. Subfigure (**e**) shows the population differentiation of experimental series, derived through hierarchical clustering on the pairwise F_st_ statistic, calculated using integrated genotypic data, with the differentiation displayed on the x-axis

The genotypes in our Big Data represent mostly elite Central European winter wheat breeding material, developed by over 14 different wheat breeding companies. Phenotypic data for both lines and hybrids were collected through public–private partnership projects during the sowing seasons from 2009 to 2021. These projects are organized into seven experimental series (Exp_1 to Exp_7) and include 98,175 grain yield data points across 31 different sites (Fig. 1a), as derived after accounting for experimental design effects at 117 environments, i.e. trial-site-year combinations. The material tested in these environments was characterized using microarray-based genotyping for 9,797 single nucleotide polymorphism (SNP) markers (see Methods).

We examined the quality of the phenotypic data for environments with replicated data (~ 51% of total environments) and observed predominantly high repeatabilities for grain yield (median = 0.81) (Additional file 1: Table S1). Since part of the environments had non-replicated trials, we estimated the genomic repeatabilities, using the GBLUP model [4], and confirmed the high quality of the phenotypic data (Additional file 1: Table S2).

The seven experimental series were linked by up to 37 overlapping genotypes (Additional file 1: Table S3), facilitating an integrated phenotypic data analysis of the 6,766 unique lines and 6,519 unique hybrids. These overlaps were detected following a genomic deduplication step where genotypes with Rogers’ distance values < 0.03 were identified as duplicates. We derived best linear unbiased estimates for line and hybrid genotypes by correcting for environmental effects and found that hybrid genotypes outperformed their line counterparts across Exp_1 to 5 (Additional file 2: Fig. S1). Broad-sense heritabilities were high, with estimates of 0.89 for lines and 0.87 for hybrids (Additional file 1: Table S4). For both, the variances due to genotype times environment (G × E) interactions were significant (*P* < 0.05) and approached 33% of the genotypic variance for lines and 43% of the genetic variance for hybrids. The variance of the G × E interactions was likely underestimated, when compared to values observed in German registration trials [23], due to the non-orthogonal design including non-replicated trials. Nonetheless, the underlying data are well suited to explore the potential of new genome-wide prediction models in modelling G × E interactions.

To gain a first glimpse into the climatic diversity of environments, we used environment variables such as precipitation, temperature, and solar radiation (Additional file 1: Table S5), which were recorded daily during the growing season from October of the sowing year to August of the harvest year. Monthly means of these variables over the growing season were used to characterize 103 site-year combinations. This number is lower than the total number of environments since multiple trials were conducted side by side in few site-year combinations—such trials shared the same climate data. We then estimated an environment relationship matrix (${ERM}_{l}$; see Methods). Cluster analysis based on the first two principal coordinates of this matrix revealed a wide diversity among environments (Fig. 1b, c). The analyses also provided a clear indication that the effects of years, and possibly their interactions with the sites, contribute to the similarity between environments.

### Lack of pronounced inter-experimental series differentiation for elite lines

Line genotypes in Exp_1 to Exp_5 include parent lines used to produce single-cross hybrids (Additional file 1: Table S1), as well as released varieties used as controls to link trials within their respective experimental series, with one exception. The male parents in Exp_5 include 360 gene bank accessions, i.e. plant genetic resources (PGRs), from the *Federal *Ex situ* Gene Bank* hosted at the *Leibniz Institute of Plant Genetics and Crop Plant Research*, Germany (IPK). Exp_6 and Exp_7, on the other hand, comprise solely of lines and released varieties used as controls. The genetic differentiation of the PGRs from the elite breeding pool is clear both in the principal coordinate analysis (Fig. 1d) and in a cluster analysis based on F_st_ statistic [24, 25] (Fig. 1e, Additional file 2: Fig. S2). In contrast, the elite lines used in the different experimental series showed only weak population structuring. It is therefore expected that the genome-wide predictions across distinct experimental series are only slightly influenced by population differentiation.

The genetic structure of hybrids mirrors the structure of their parent lines, with hybrids between PGRs and elite lines from Exp_5 forming a distinct and separate cluster compared to the elite by elite crosses from rest of the series (Additional file 2: Fig. S3). It should be noted that most hybrids have been produced on the basis of incomplete factorial mating designs, which results in a much higher degree of relatedness between hybrids within experimental series than between hybrids from different experimental series (Additional file 2: Fig. S4).

### Data volume reaches lower limit for competitive grain yield predictions based on artificial intelligence

**Fig. 2:**
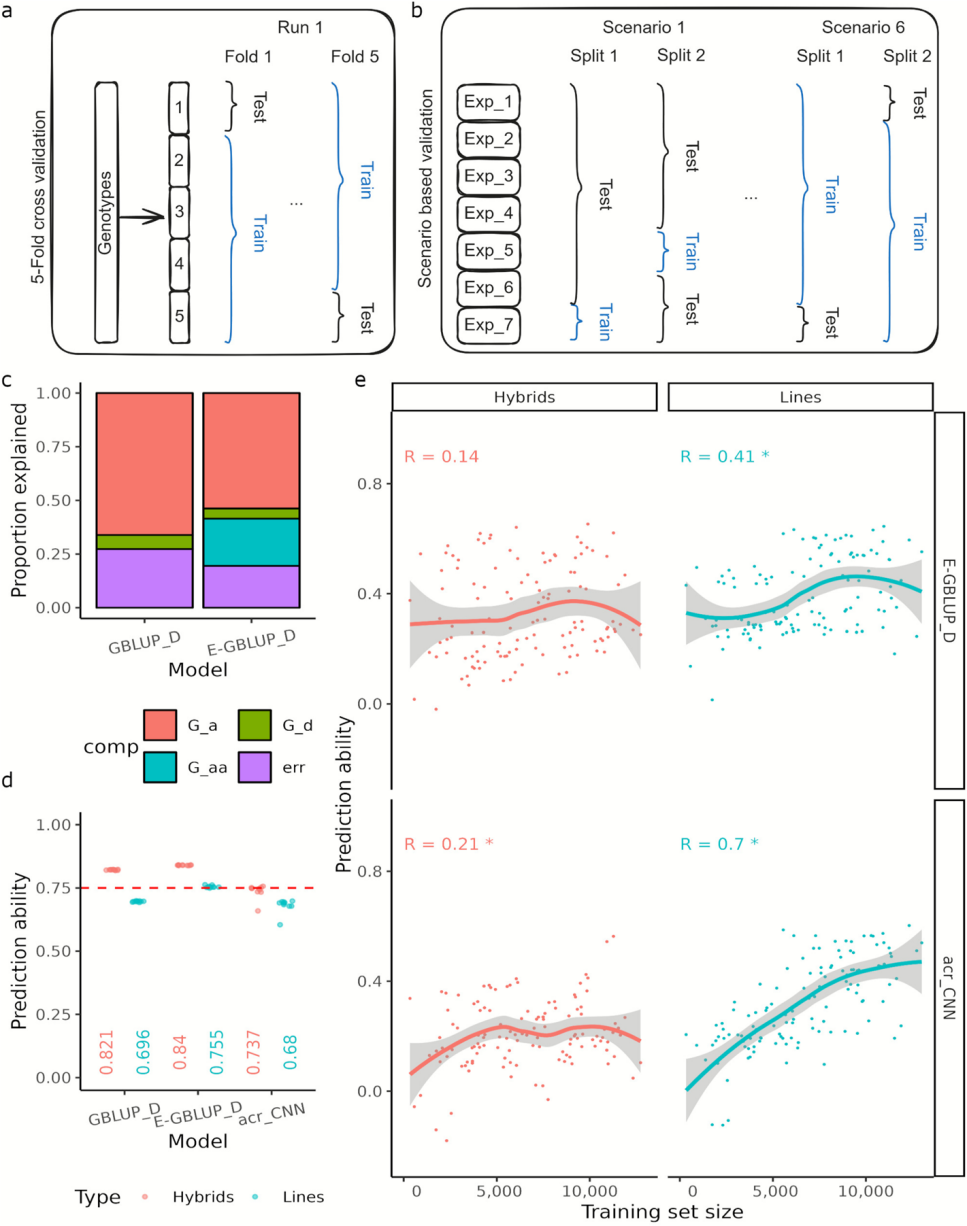
Overview of prediction of average genotype performance. Schematic of (**a**) five-fold cross-validation: genotypes were split randomly into five folds for a given run, and grain yield for one-fifth of the genotypes was predicted using the remaining genotypes as the training set, (**b**) Scenario based validation: Genotypes were split according the experimental series they belonged to: in scenario 1, the training set consisted of a single experimental series; in scenario 6, it comprised a set of six randomly selected experimental series for each split, (**c**) Proportion of total phenotypic variance explained by the genomic additive (G_a), dominance (G_d), and additive epistatic (G_aa) components. Genomic prediction abilities when predicting average genotype performance under: (**d**) five-fold cross-validation–with GBLUP_D, E-GBLUP_D and acr_CNN, (**e**) Scenario based validations–with E-GBLUP_D and acr_CNN. The lines were fit with LOESS (locally estimated scatterplot smoothing) function, regressing mean correlation(s) on training set size. “*” indicates that Pearson correlation coefficient (R) between training set size and mean correlation is significant at p-value threshold of 0.05. Results are shown separately for hybrids and lines

The following analyses are based on the best linear unbiased estimates of grain yield performance for line and hybrid genotypes, estimated across environments using model (1) (see Methods). Two validation types were employed: (a) five-fold cross validations (Fig. 2a and b) scenario-based validation (Fig. 2b). With the former, two GBLUP [4] based models were tested: GBLUP_D model accounting for additive and dominance marker effects and E-GBLUP_D model additionally accounting for additive epistatic effects with the genotypic data. Due to the different relationships between lines and hybrids, we analyzed both groups separately, although the relevance of hybrid-specific dominance effects for our data was low (Fig. 2c): Dominance effects explained only 4.7% of the phenotypic variance, whereas the contribution of additive and additive times additive epistatic interaction effects were higher, explaining 53.7% and 22.1% of the same, respectively.

The relevance of epistasis was also confirmed by evaluating prediction abilities using five-fold cross validation (Fig. 2d). Here, E-GBLUP_D led to an average increase in prediction ability of 2.3% and 8.4% for both hybrids and lines compared to GBLUP_D. A deep learning model, acr_CNN–designed with a variable stack of convolutional layers followed by dense layers (Additional file 3: Material S1)–was also evaluated. While acr_CNN did not outperform E-GBLUP_D, it was competitive with GBLUP_D for predicting line performance, achieving a mean prediction ability of 0.68 versus 0.7 for GBLUP_D (Fig. 2d). Due to the close relationship between the hybrids of the training and test populations resulting from the application of five-fold cross validation, the prediction ability of the hybrids in the case of E-GBLUP_D was 11.2% higher than that of the lines.

The picture changed with scenario-based validations in which we ensured that certain experimental series were used in either the training or the test set and also gradually increased the size of the training set. Six scenarios were considered which differed regarding the number of experimental series in the training set. In scenario 1, the training set included one experimental series, while in scenario 6, the training set comprised a stack of six randomly selected experimental series. Using successive scenarios, the relatedness between training and test sets was reduced, mirrored by increases of mean pairwise genetic distances up to 23.81% for hybrids and 17.28% for lines in comparison to the within series values (Additional file 2: Figs. S4 and 5). The penalty for this reduced relatedness was a halving of prediction ability for both hybrids and lines (Fig. 2e), when examined against comparable training population size as used in five-fold cross validation. Nevertheless, we observed an interesting trend for the line group: While the prediction abilities for E-GBLUP_D plateaued with larger training sets, a steady increase was observed for acr_CNN over the range of training set sizes investigated (Fig. 2e). In scenario 6, for example, the acr_CNN outperformed E-GBLUP-D in 4 of the 7 splits. Given the non-cross validated nature of the modified study, we additionally assessed acr_CNN competitiveness as splits in a given scenario where the difference in model performance was at most 0.06. Notably, acr_CNN performance for lines rose consistently from 14 to 86% of splits from scenario 1 to 6 (Additional file 1: Table S6).

### Modeling G × E interactions with Big Data enables enviromically adapted predictions

**Fig. 3:**
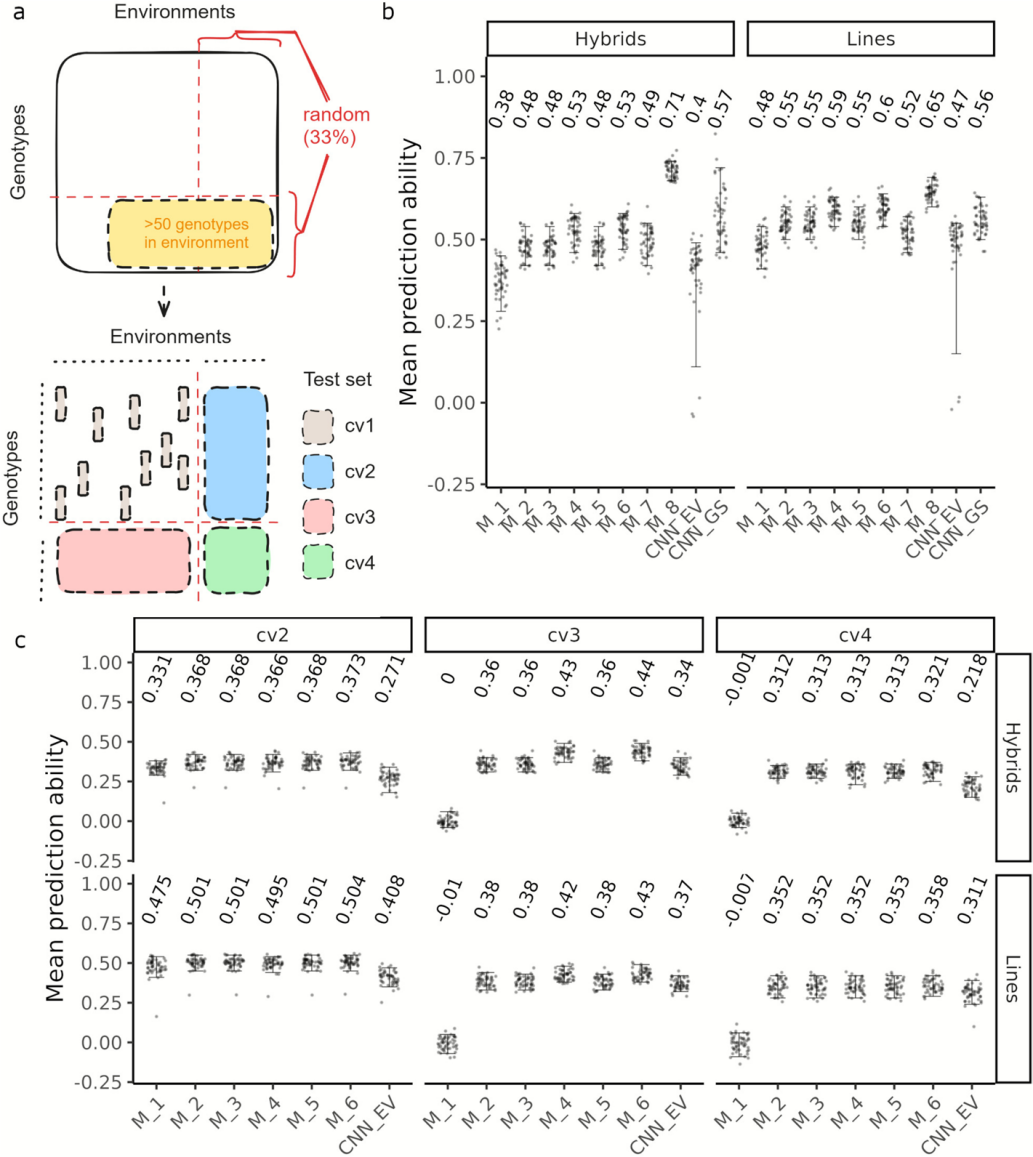
Overview for enviromically adapted genomic predictions. Schematic of (**a**) cross-validation scheme: As a first step environments with more than 50 genotypes were filtered. The remaining data were then split into four quadrants (shown by intersecting red lines) with fourth quadrant (bottom right) containing 33 percent of unique dataset genotypes in 33 percent of unique dataset environments. A given run comprised cv1 to cv4. Test set for cv1 was a 20 percent random sample from quadrant 1, while complete quadrant 2,3 or 4 was used as test sets for cv2, cv3 and cv4, respectively. The training set for a run was the remaining 80 percent sample from quadrant 1. Mean prediction ability for grain yield with cross validation scenarios (**b**) cv1 and (**c**) cv2, cv3 and cv4. Results are shown separately for hybrids and lines. Error bars represent 5th to 95th percentile range

Sparse genotype testing often results in incomplete multi-environment phenotyping data, where some genotypes are only evaluated in subset(s) of the total environments. We created this scenario using cv1 (Fig. 3a) and employed genomic prediction to fill the data gaps. For this, we defined the baseline linear mixed model M_1 which incorporated genotype and environment main effects with a design matrix of '0's and '1's to associate phenotypic records with the corresponding effects (see Methods for an overview). All cross-validations were performed using grain yield data from a reduced set of 54 out of 117 environments. This data were only corrected for experimental design effects and involved approximately 10,000 genotypes for which extended information on soil characteristics, crop management, and heading date was available (Additional file 3: Material S2).

With M_1, we observed mean prediction abilities of 0.38 for hybrids and 0.48 for lines. Substituting the main genotype effects with additive, dominance, and additive epistatic effects, similar to E-GBLUP_D, in model M_2 improved these values up to 29% for hybrids and 15% for lines (Fig. 3b). However, further inclusion of linear or non-linear environmental effects in models M_3 and M_5 did not enhance prediction performance beyond M_2. This indicates that simply considering environmental effects as such is not informative for predicting genotype performance.

We therefore extended models M_3 and M_5 to account for genotype times environment (G × E) interactions with a multiplicative effect in models M_4 and M_6. The variance–covariance structure of the G × E effects was derived as a point wise “$\odot$” Hadamard product between additive genomic and respective environment relationship matrices. These models captured 74.3% of the baseline genetic variance (Additional file 3: Material S3) and led to a further improvement of up to 12% for hybrids and 8% for lines over model M_2, with model M_6 being slightly better than M_4 (Fig. 3b).

In scenario cv2, we predicted the performance of genotypes in new environments not included in the training set. Here with M_1, we recorded a mean prediction ability of 0.33 for hybrids and 0.47 for lines. Model M_2 improved the prediction ability up to 12%, while model M_6 provided only a slight additional increase of 2% over M_2 for hybrids (Fig. 3c). However, the latter was not statistically significant according to a paired samples t-test. Clearly, benefit from modelling G × E interactions depends on whether the genotypes to be predicted have been partially evaluated in the training set environments.

### Model performance in predicting new genotypes decreases with training-test set relatedness

A breeder is primarily interested in predicting the performance of new, improved genotypes without having to test them in the field. We developed two potential scenarios mimicking this situation: one predicting the performance of new genotypes in environments where other genotypes have been tested (cv3), and another predicting their performance in entirely new environments (cv4). In both scenarios, predicting genotype performance with M_1 was not possible (mean prediction ability of 0) because no genomic or environment relationship was exploited. Accounting for the former with M_2 resulted in mean prediction abilities of 0.38 and 0.36 for lines and hybrids, respectively, for cv3 (Fig. 3c). Model M_6, which accounted for G × E interactions, further improved prediction abilities by up to 23% for hybrids and 12% for lines. For cv4, only model M_6 showed an improvement over M_2, increasing prediction ability for hybrids, for example, from 0.31 to 0.32, a 3% improvement. The marginal improvements conferred by M_6 in cv4 can be attributed to the increased complexity in modelling G × E interactions, in addition to the sharp decrease in training-test set relatedness.

### Neural network performance scales poorly with network complexity

To address the non-linear complexity in modelling G × E interactions, we developed a deep learning based genomic prediction model CNN_EV. This model, similar to acr_CNN, is a dynamic model that tunes its architecture to best abstract the relationship between training and validation set based on additive genomic and environment variables (EV) information (see Methods, Additional file 3: Material S4). Across all cross validation scenarios, i.e. cv1 to cv4, moderate to low prediction abilities were realized with CNN_EV (Fig. 3b, c). This is in sharp contrast to the trends observed when using these for predicting average genotype performance in lines (Fig. 2). One potential reason for this discrepancy could be the way input data were represented. In this study, separate CNNs were used for (additive) genomic and EV data, whereas for the latter other studies have employed either raw or aggregated data directly in CNNs [16] or in long short-term memory (LSTM) neural networks [26]. Additionally, CNN_EV may not have fully captured G × E interactions, as they were designed to learn non-linear G × E interactions through high-dimensional feature representations rather than explicit inputs. Interestingly however, for cv2 and cv4 CNN_EV was the best performing model based on root mean square values (Additional file 3: Material S5).

During model tuning, the hyperparameter space was set for maximum flexibility, resulting in models with over 205 million parameters (Additional file 3: Material S4). Despite this high level of complexity, these models were less effective than their GBLUP-based counterpart (model M_6). This suggests that increasing model complexity does not necessarily improve prediction performance and that G × E interactions need to be translated into informative features to better understand their role in the expression of crop phenotype.

### Characterizing genotype responses to growth environment is informative for filling gaps in unbalanced data

We used the crop growth model MONICA to parameterize the growth of genotypes in the data from the previous section(s) at specific sites. MONICA captures non-linear physiological responses to environmental conditions that affect plant growth and contribute to grain yield. Initially, model parameters corresponding to phenological stages were determined followed by those describing biomass accumulation and yield formation (Additional file 1: Table S7).

When combined with genomic, environment and multiplicative G × E interactions information (model M_8), the inclusion of these parameters resulted in mean prediction abilities up to 45% higher than those observed with model M_7 (Fig. 3b), which modeled genotype-site effects using a design matrix of only '0's and '1's in the cv1 cross validation scenario. M_8 also outperformed M_6 with improvement in mean prediction abilities of up to 34% for hybrids and 9% for lines. Adapting CNN_EV to work with MONICA-derived parameters (model CNN_GS) could not outperform M_8 but resulted in improvements of up to 47% for hybrids over CNN_EV. Evidently, by parameterizing genotype-specific physiological responses at each site, we essentially decompose the G × E interaction into mechanistic components that can be more accurately predicted–in Additional file 3: Material S3 we show that G × E variance decreases from M_7 to M_8. It must be noted however, that due to limited data availability, MONICA parameters were not derived for each training-test split, the prediction abilities achieved with M_8 therefore represent an upper limit of predictive performance.

Since MONICA parameters could potentially capture G × E interactions, we explored the genetic component for these using the training-test split of cv3 cross validation scenario. Genomic and error variances for MONICA parameters were estimated for each environment within a given training-test split using the GBLUP model [4]. Genomic repeatabilities were then calculated, with most parameters showing low values (~ 0.15, Additional file 2: Fig. S6). However, base temperatures at all phenological stages (BaseT*) and temperature sums at emergence, double ridge and heading (Tsum-1, 2, & 3), had higher values (~ 0.29). We interpret the missing repeatabilities as indicative of a significant G × E interaction component and suggest that while model calibration is data-driven, some parameters may be predicted with genomic data, with the rest may be learned during model calibration.

Lastly, we implemented a specialized model for predicting grain yield using genomics-assisted crop growth modeling (model CGM_only) in a leave one environment out cross validation scenario (LoO). For this we used MONICA calibrated with training set information to predict grain yield for the test set comprising single environments. Notably, CGM_only fared poorly compared to other models (Additional file 2: Fig. S7), possibly due to model calibration relying on data from sites that were different from those in the test set. Highlighting clearly the need to rethink the design of plant breeding experiments and data sharing to better exploit such frameworks.

### Groups of environments with similar G × E patterns could be partially explained by environment variables

**Fig. 4:**
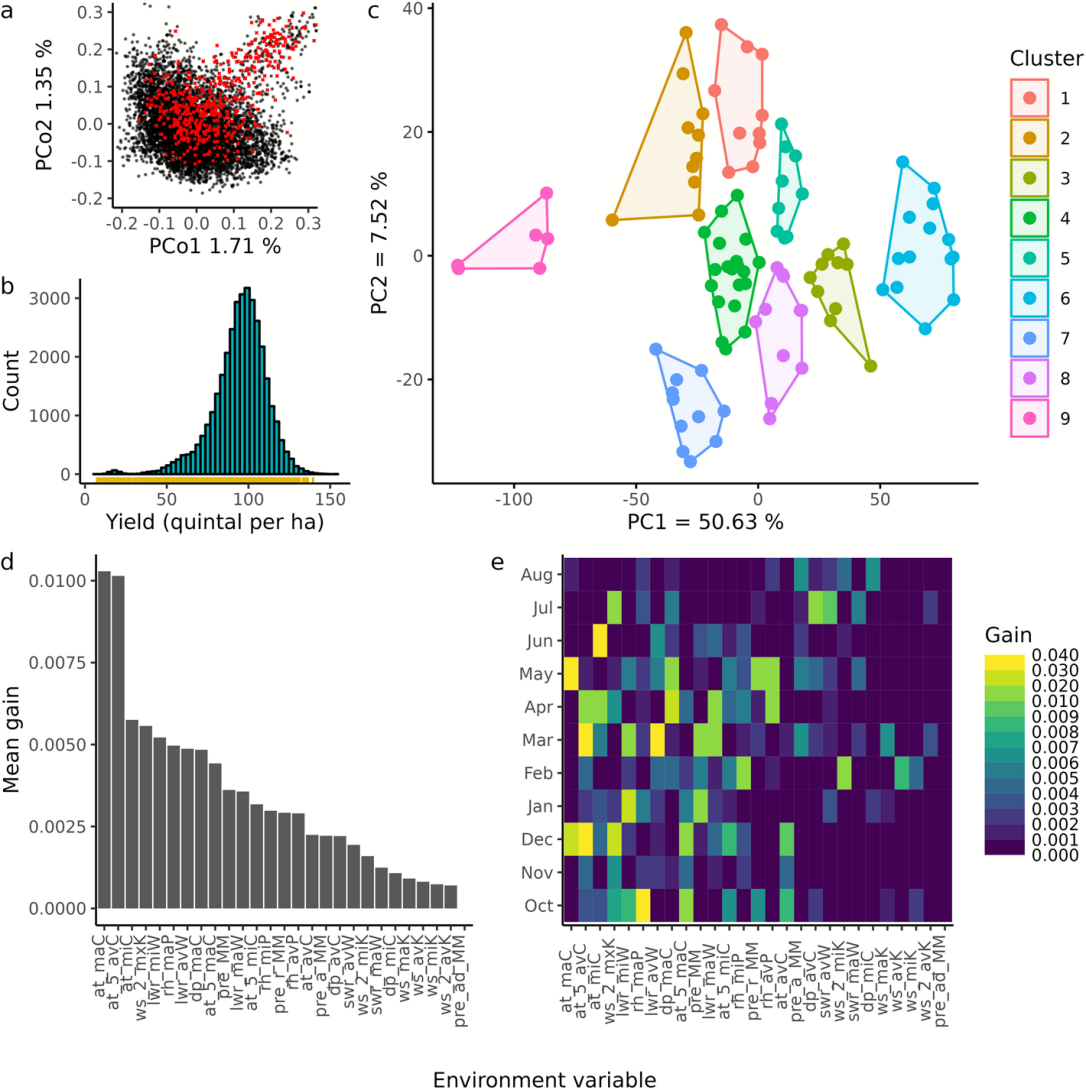
Characteristics of core set: (**a**) genetic diversity space of core set (red) against remaining line genotypes (black) with principal coordinate analysis (PCo) plot of Rogers’ distance matrix, (**b**) grain yield distribution of core set (rug plot at bottom) and that of remaining genotypes (histogram). Cluster analysis: (**c**) environment clusters with number of clusters set to nine. PC1 and PC2 represent the first two principal components of Euclidean distance matrix derived using G × E patterns. Feature importance analysis: (**d**) Environment variables ranked in descending order based on mean scaled gain values calculated across growth periods, (**e**) heat plot of scaled gain values across growth periods and environment variables. The abbreviations of the environment variables are explained in Additional file 1: Table S5

To better understand the model benchmarks, we shifted our attention to examine the nature of G × E interactions in our data. For this, we selected a core set of 500 lines to represent a large proportion of the genetic (red crosses in Fig. 4a) and phenotypic diversity (Fig. 4b; Additional file 1: Table S8). Using model M_6, we predicted the performance of the core set in all 117 environments to examine the relationship between tree clusters based on G × E pattern information (right tree in Additional file 2: Fig. S8) and those based on environmental variables (Fig. 1c, also on the left in Additional file 2: Fig. S8). The G × E pattern was defined as the residuals from the predicted values that could not be explained by genotype or environment main effects, thus capturing the specific response of genotypes to different environments. This was possible since climate data that were available for all environments were sufficient for model M_6.

Despite the low similarity between the trees, with a cophenetic correlation coefficient of 0.26, there was a clear trend toward strong sub-tree identity, indicating that the core set's performance was responsive to a change in environment. We then identified nine clusters, each containing at least 6 environments with similar genotype times environment (G × E) patterns. (Fig. 4c) using K-means clustering [27], a machine learning approach. When projected onto the map of Europe, the environment clusters showed only weak geographical but strong seasonal patterns (Additional file 2: Fig. S9), suggesting that the group a site belongs to may vary with year. We conclude that the year effect, rather than fixed site characteristics, may be the major driver of G × E interactions.

To capture relationships between observed G × E pattern and environment variables (EVs), we used a gradient boosting tree classifier [28], a machine learning approach and show that the EVs can at least partially explain the clustering: on average 62% of the test set environments were correctly classified based on EVs in a validation study. Air temperature (at_maC, at_5_avC, at_miC), wind speed (ws_2_mxK) and long wave radiation (lwr_miW), were found to be important factors driving G × E interactions (Fig. 4d) based on “gain” importance scores. The "gain" scores measure the improvement in accuracy that a feature brings to the tree branches in which it is used (https://xgboost.readthedocs.io/en/stable/index.html, release 1.7.0). Interestingly, the relevance varied within a season i.e. year, with relative humidity (rh_maP) during emergence (middle of October) and air temperature during tillering (end of November to December as well as March) as well as heading (May to June) being the most important influential variables affecting crop growth (Fig. 4e). Combined with the influence of temperature at specific phenological stages, these results confirm that temperature plays a significant role in crop growth.

### Enviromically adapted genotypes identified with Big Data to increase grain yield productivity

Based on the best linear unbiased estimates of grain yield performance for line and hybrid genotypes, estimated across environments, we curated a reference set of 675 lines. This set included subset 1 of 473 lines with grain yields above 101 Q ha^−1^, and subset 2 of 202 lines with grain yields ranging between 60 and 100 Q ha^−1^ (Additional file 2: Fig. S10). Using model M_6, we predicted the performance of the reference set in all 117 environments and identified enviromically adapted genotypes, focusing on the best performing 10% out of the 675 lines in each environment.

**Fig. 5:**
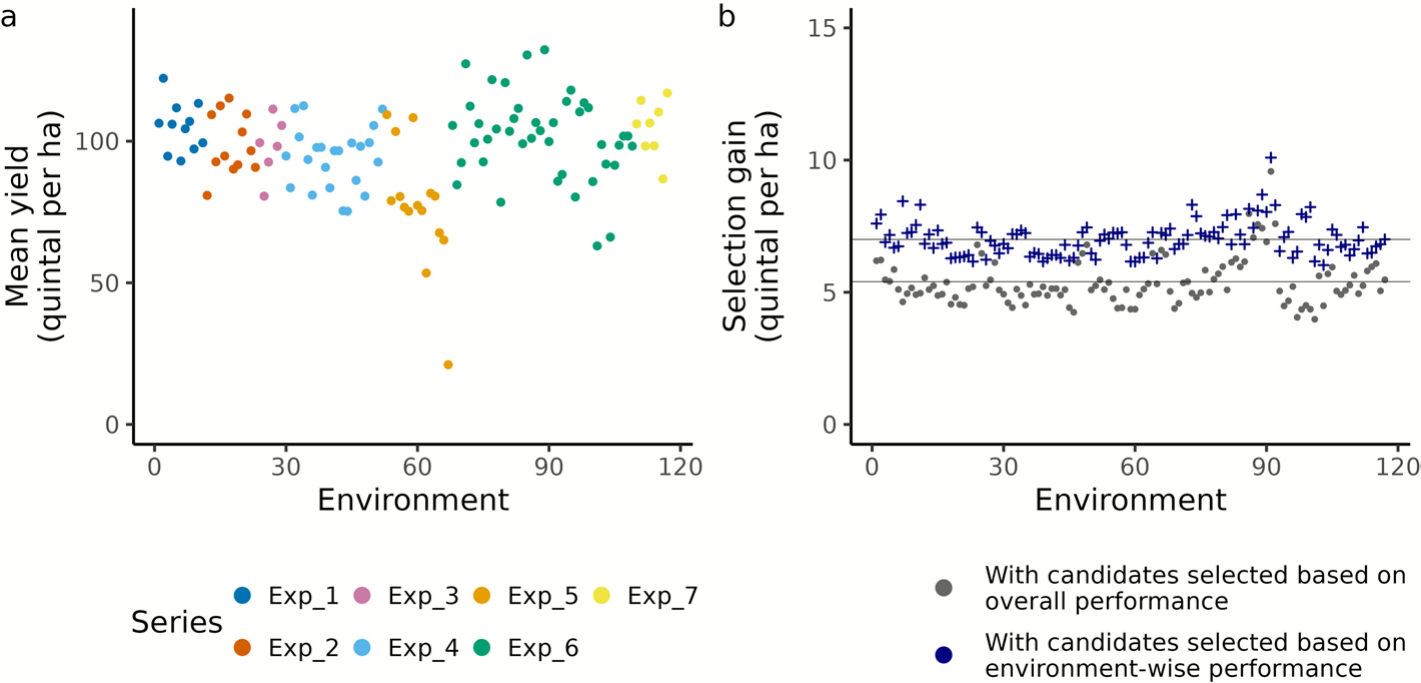
Overview of competitiveness: (**a**) environment-wise mean (predicted) yield of the reference set across 117 different environments. These environments are color-coded based on the experimental series they belong to. (**b**) selection gain (expressed as the yield difference) relative to the environment-wise means for the reference panel. This gain is reported for the enviromically adapted genotypes, defined as the top 10% of high-yielding genotypes in each environment, as well as for the 50 overall high-yielding performers from the reference set

Interestingly, the mean performance of reference set varied greatly between environments (Fig. 5a), with values ranging from 21.09 Q ha^−1^ to 132.28 Q ha^−1^. These values fall well outside the range of their best linear unbiased estimates used for curation, underscoring the strong influence of G × E interactions on genotype performance and the importance of considering them in selection decisions. We further assessed the performance fluctuations of enviromically adapted genotypes within nine environment clusters, previously identified with the core set. Notably, these were more often identical within the same cluster (averaging 38 out of 67) than across different ones (averaging 29 out of 67), with a few exceptions (Additional file 1: Table S9). This points to exploiting G × E interactions in clusters of environments. In practice, however, the choice of varieties is mainly determined by site characteristics. Therefore, the clusters would need to be adjusted for the year effect to derive broader target regions, i.e. combinations of different sites.

Currently, regional recommendations favor genotypes selected based on average performance, i.e., best linear unbiased estimates. We identified the 50 overall (averagely) performing genotypes from the reference set and evaluated their yield productivity as a baseline for traditional breeding pipelines. The predicted yields of these genotypes showed a mean selection gain of 5.37 Q ha^−1^ (Fig. 5b) above the environment wise mean(s) for the reference panel (Fig. 5a). This highlights the importance of testing the performance of genotypes before recommending them for national listing [2]. Importantly however, enviromically adapted genotypes achieved higher selection gain, with a mean value of 7.02 Q ha^−1^(Fig. 5b). When compared to the overall performers, the latter is a boost of up to 3.86 Q ha^−1^ (mean = 1.65 Q ha^−1^), with a minimum of 0.18 Q ha^−1^ depending on the environment. With an average genetic yield gain of 0.32 Q ha^−1^ year^−1^, which has been estimated for winter wheat in Germany [22], the yield boost corresponds to up to 12 years of realized breeding progress. Clearly, there is a lot of hidden yield potential in breeding programs that needs to be translated into candidate recommendations.

## Discussions

In our study, we investigated the potential of genome-wide predictions for the selection of enviromically adapted varieties when integrating public and private datasets in the form of Big Data. The potential gain in on-farm yield due to improved prediction ability–when accounting for genotype times environment (G × E) interactions for the key trait grain yield in cv1 as well as cv3 cross validation scenarios–is promising. Many factors contributed to these results, such as successful data curation (Fig. 1) and benchmarking of different modelling frameworks (Figs. 2 and 3) in particular the use of crop growth model MONICA to integrate environment variables for genome-wide predictions.

Amongst the models compared for predicting mean genotypic values within environments, clear trends emerged across cross validation scenarios cv1 to cv4 (Fig. 3). Models M_4 and M_6, both accounting for G × E interactions, consistently outperformed models M_1 to M_3 and M_5. Between the former, M_6 showed a marginal advantage, likely due to its ability to account for high order G × E interactions. Further, with model M_6, we observed that the hybrid group benefitted the most in terms of improved prediction abilities, although the line group had higher base values to start with. We identified scope for further investigation into cv2 and cv4, requiring prediction of genotype performance in new environments, where marginal improvements were recorded with model M_6, a trend mirrored by the corresponding root mean square error (RMSE) values (Additional file 3: Material S5). Higher prediction abilities may be realized by shifting these to ideally enable cv1-like sparse testing scenarios [9, 29, 30]. This calls for a paradigm shift in how plant breeding experiments are designed. However, once implemented, it would enable a transition from the traditional method of selecting varieties based on overall high performance to selecting enviromically adapted varieties.

A crucial component for this is the availability of climate data, often ignored during the course of a breeding program. For instance, we achieved improvements of up to 23% in predicting the yield of new hybrid genotypes within the cv3 cross validation scenario (Fig. 3). While one way to collect such data is by installing weather stations at trial sites, another option, though with a coarser resolution, is to use publicly available long-term climate data collections, or data derived from optical satellite sensors [31]. Notably, informative environment variables are likely to vary depending on the trait of interest [32], the method of gridding i.e. whether based on crop phenology or time period wise aggregation, the diversity of sampled sites amongst others, although some overlap may be expected. For instance, our findings (Fig. 4) partially align with those for maize [33], indicating that temperature-associated environmental variables are the most informative for yield prediction. Predictive breeding can therefore benefit by incorporating weighted environmental variables into relationship matrices, analogous to weighted genomic relationship matrices [34].

Notably, access to climate, soil and management data allowed parametrizing genotype growth with crop growth model MONICA, which translated into highest predictive performance by model M_8 in cv1 cross validation scenario. M_8 outperformed M_6 due to: (1) the decomposition of G × E into genotype times site (G × S) and genotype times year (G × Y) components, and (2) robust estimation of G × S effects through MONICA derived parameters. Extending M_8 to cv3 like scenarios is not possible since MONICA cannot be calibrated for new, previously untested genotypes. One possibility however could be to predict the MONICA derived parameters using genomic information. This would also require restricting the parameter space to be estimated since not all parameters exhibit a high genetic component (Additional file 2: Fig. S6). As a practical starting point, models like M_6, which require genotypic, phenotypic, and climate data, are more broadly applicable—especially within the trial network of a single breeding program, where environment-specific rather than broadly superior genotypes may be prioritized early on.

Eventually, moving outside a breeding program will be needed to capture more sites and therefore benefit from a better estimate of G × E interactions. In our Big Data for instance, Exp_6 represents data from such a breeding program designed for line genotypes only. Fusing the latter with public data, from Exp_1 to 5, enabled investigations into genomic predictions for winter wheat hybrids. Another possibility for such an effort would be to utilize data from public variety testing trials. In Germany, for example, every three-year release cycle, around 120 post-breeding candidates are evaluated at 35 to 40 sites [23]. Of these, only 15 to 20 genotypes are further evaluated in regional trials, based on which recommendations are made to farmers. While a simulation framework can be used to assess the performance of recommended varieties [12], it cannot compensate for missing genetic diversity in the training data. This makes integrating public variety testing trial information into company data silos [35] even more lucrative, despite being legally and technically complex. Breeding companies may however enter into crossing agreements for early use and genotyping of the tested candidates to eventually inform their breeding programs with information from public variety trials. Redesigning the latter with more candidate varieties that are not tested orthogonally [36] would also be advisable to go beyond lower bounds of data volume (Fig. 2) for genome-wide predictions and adoption of deep learning methods, but would certainly require intensive discussions and research into hyperparameter tuning. The latter was the most time-consuming component for convolutional neural network models (acr_CNN, CNN_EV and CNN_GS), accounting for up to 90 percent of the compute time in some runs. Overcoming the data bottleneck will likely benefit CNN_EV, which showed low RMSE for cv2 and cv4 (Additional file 3: Material S5): as prediction abilities become comparable to those of GBLUP-based models at higher data volumes, the lower RMSE suggests that CNN_EV may provide less biased estimates of breeding values. Future work should implement multiple replicate validation schemes with different random seeds for train-validation splits to reduce generalization error and better account for the prediction outliers observed in our CNN models.

Breaking company data silos for integrating data could widen the genetic diversity typically available within a breeding program and can inform genomic prediction models [37]. This integrated use of data could also help compensate for the internally unbalanced nature of the data, benefiting various actors in the breeding process. However, such data sharing requires a harmonized plant breeding data ecosystem and the implementation of a data trustee model to manage the rich data from the breeding industry [38]. Successful data sharing models in cattle breeding [39] as well as human genomics research [40] show that such models are not necessarily a pipe dream, though they cannot simply be copied in plant breeding. This is chiefly due to the lack of, and limited adoption of, comprehensive ontologies [41], along with challenges such as upgrades to computational infrastructures and issues with data quality.

In this study, we used an in-house computing cluster consisting of several servers to support our analysis (Additional file 1: Table S10). This infrastructure significantly accelerated computation, particularly for memory-intensive models such as M_6, which required up to 200 GB of computing memory. We also leveraged graphics processing unit acceleration to efficiently run convolutional neural network-based prediction models. Such computational resources are not commonly employed by small- to medium-sized breeding companies. While cloud-based alternatives (e.g., Amazon Web Services) are available, their long-term operational costs can surpass those of maintaining a dedicated local setup, especially for large-scale, routine analyses. However, the value of such high-performance infrastructure ultimately depends on the quality of the data being processed. Our work builds on years of experience integrating fragmented genotypic and phenotypic datasets, with a focus on evaluating their alignment with key estimates such as environment-based (Additional file 1: Table S1) and genomic repeatabilities (Additional file 1: Table S2). High genomic repeatabilities point to low imputation-derived errors, in line with previous studies [37], and pave way for genomic deduplication step to derive BLUEs across environments [20]. These assessments, grounded in ontologies, are essential for ensuring data quality and must precede major investments in computational infrastructure to avoid the "Garbage In, Garbage Out" effect.

The above considerations are based on data from breeding experiments and depend heavily on the representativeness of the experimental sites for the entire set of target environments. Ultimately, the predictions must be relevant to farmers, who encounter specific growth conditions at their sites. Clusters of similar environments can serve as a first approximation for the same and may be derived from harmonized data (Fig. 4). In a second step, genome-wide predictions can be used to help farmers identify superior varieties for target clusters overlapping with the site of their interest (Fig. 5). Since year-to-year fluctuations at a site also contribute to G × E interactions, it is advisable to finetune the identification of superior varieties by selecting ones with higher yield stability, informed by historic Big Data [12].

Lastly, if farmers, in turn, contribute their crop data, including details of the varieties grown and crop management information, the prediction models can be iteratively improved [42, 43]. This will certainly require a radical rethinking in Europe, but it would help to close the yield gap in wheat production [44].

## Conclusions

As plant breeding embraces the digital age, great opportunities open up to exploit diversity of data types and bolster decision making towards higher genetic gains. However, a major challenge lies in identifying the right data types and sources, and most importantly, ensuring data access and interoperability. In this study, we propose a genomic prediction framework that addresses these points by fusing data from public–private sources and predicting performance of enviromically adapted genotypes. In future, these analyses should be expanded by focusing on additional key traits with economic and social value, such as disease resistance. Such efforts will facilitate exploration of the fragmented data ecosystem and thereby translate breeding progress more effectively to farmer fields.

## Methods

### Phenotypic data

This study was based on existing wheat grain yield data [20] corrected for the statistical effects of the field trial designs [45] (Additional file 3: Material S6), across six experimental series (Exp_1 to Exp_6). Exp_1 to Exp_4 were based on phenotypic data for single-cross hybrids, their parents and a few released varieties, all adapted to Central Europe. Exp_5 included phenotypic data from hybrids produced by crossing elite winter wheat lines with historical varieties or accessions from the *German Federal *Ex situ* Gene Bank*. The accessions were selected after screening gene bank material for pronounced anther extrusion and the historic varieties, characterized by short plant height, originated from all over Europe over the last four decades. In addition to the hybrids, their parents and released varieties were also tested. Exp_6 was based on Central European elite winter wheat lines from the breeding program of KWS Lochow GmbH (Bergen, Germany). Additionally, for Exp_6, phenotypic data were extended to include two years (sowing years 2021 and 2022) of field trials for advanced winter wheat lines in late selection stages from KWS Lochow GmbH (Additional file 3: Material S7). Exp_7 included data from the GABI-WHEAT [46] panel, which was compiled from European elite varieties released between 1975 and 2007 [47]. A summary of all the series is available in Additional file 1: Table S1.

**Fig. 6:**
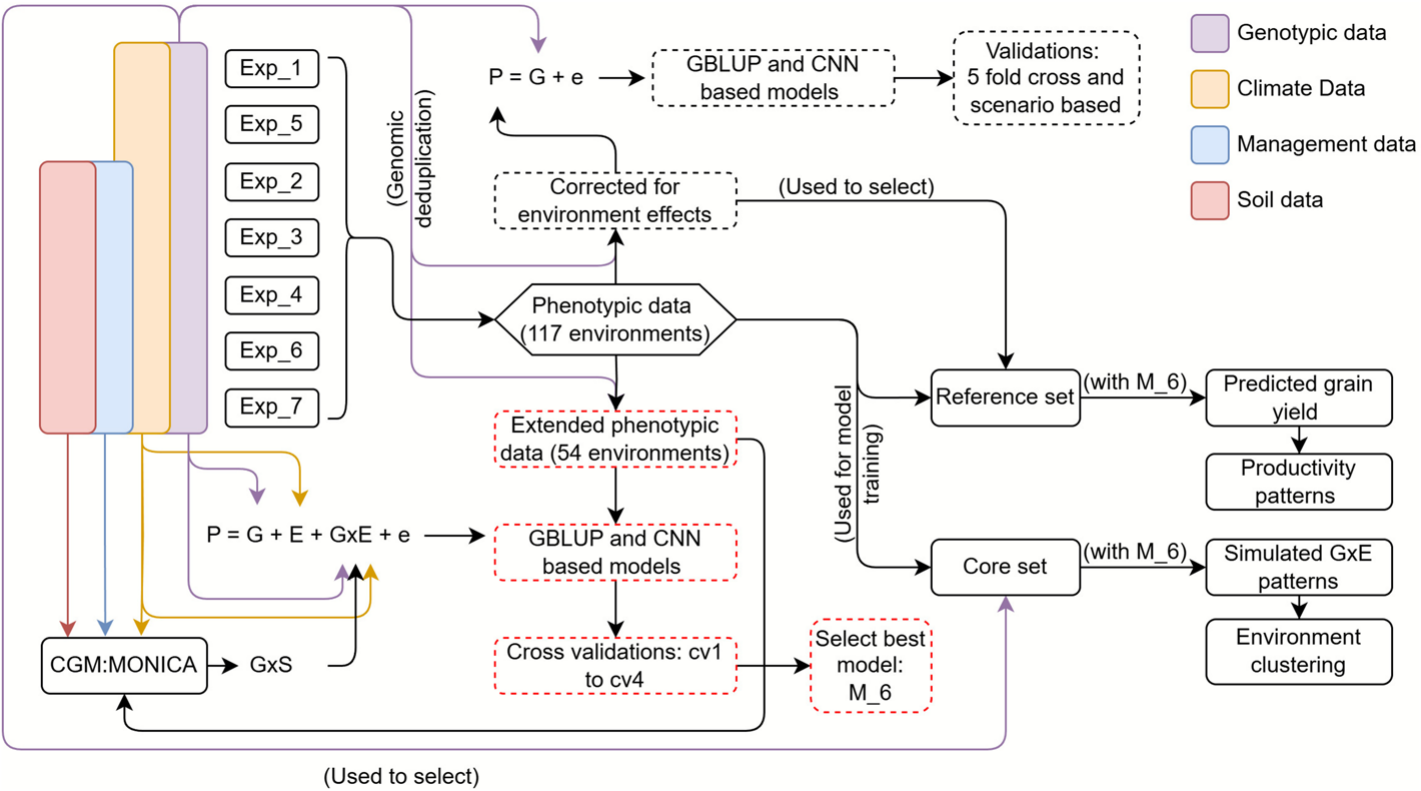
Phenotypic data (within-environment means; central hexagon) from 117 environments were curated from experimental series 1 to 7. Additional data sources included genotypic data (purple), climate data (orange), management data (blue), and soil data (pink). Climate data were available for the full set of environments, while genotypic, soil, and management data were available for 54 environments (Exp_2 to Exp_4, Exp_6, and Exp_7). Four major analyses were conducted, indicated by different box styles: (1) Upper pathway (dashed black boxes)–Genotypic data were used to identify genomic duplicates and correct the phenotypic data for environment effects, i.e. derive BLUEs. This data was then used to benchmark genomic best linear unbiased prediction (GBLUP) and convolutional neural network (CNN) models based on the equation P = G + e (phenotype = genotype + error), using five-fold cross-validation and scenario based validations. (2) Lower pathway (dashed red boxes)–Extended phenotypic data (54 environments) were used to benchmark GBLUP and CNN-based models following P = G + E + G × E + e (E = environment, G × E = genotype times environment interaction). Models were compared using cross-validation types cv1 to cv4, and the best model (M_6) was selected. Crop growth model MONICA was used to derive genotype times site interaction (GxS) information. (3) Right upper pathway–A core set was identified using genotypic data, and the complete phenotypic dataset was used as training data to simulate G × E patterns with M_6 and subsequently cluster the environments. (4) Right lower pathway–BLUEs were used to select a reference set, and the full phenotypic dataset was used as training data to predict grain yield and assess grain yield productivity patterns. Colored arrows indicate data flow by source type, with annotations provided for clarity

The curated data from the seven experimental series were integrated and analyzed (see Fig. 6 for an overview) using the following linear mixed model:

1 $$y = \mu + G\tau + Eu + e$$

where $y$ is a vector of experimental design effect corrected grain yield values ordered as genotypes within respective environments, $\mu$ is the overall mean, $G$ is the design matrix of genotypes, $E$ is the design matrix of environments, $\tau$ is the vector of genotypic effects, $u$ is the vector of environmental effects, and $e$ is the vector of residuals. To derive Best Linear Unbiased Estimates (BLUEs) for average grain yield performance, $\tau$ was assumed to be a fixed effect, whereas $u$ and $e$ were assumed to be random effects. The respective variances of $u$ and $e$ were assumed to be normally, independently, and identically distributed. For deriving estimates of genotype broad sense heritability, the following model was used;

2 $$y = Type + Series + Env + Line +{GCA}_{F}+{GCA}_{M}+ SCA+ Env:Line + Env: {GCA}_{F}+Env: {GCA}_{M}+ Env:SCA + {e}_{(Env)}$$

where $Type$ component included the specific means of lines and hybrids, $Series$ and $Env$ were effects of series and environments, respectively. The main effect of lines was modeled as $Line$ while the main effect of hybrids was decomposed into general combining ability effects of parent males (${GCA}_{M}$), parent females (${GCA}_{F}$) and specific combining ability effects of hybrids ($SCA$). The rest of the model terms refer to interaction effects between the corresponding components. The model(s) was implemented with ASREML-4.2 inside Rv4.0.5 [48] wherein all model terms except $Type$ and $Series$ were set to random, and a heterogeneous variance structure was used for residuals in each environment.

Given the highly non-orthogonal nature of the data, connectivity between genotypes in different environments was ensured following a genomic deduplication step. A Rogers’ distance matrix was calculated for all genotypes using the integrated genotypic data (see next section) and pairs of genotypes with Rogers’ distance < 0.03, indicating high genetic similarity, were considered genomic duplicates. The threshold corresponds to theoretical distance between two lines in F6 or later generation in a pedigree breeding program. The information from genomic duplicates was used to derive BLUEs. The BLUEs thus derived were mapped back to the original genotypes.

### Genotypic data

Previously defined genomic data for Exp_1 to Exp_6 [20] was expanded with additional data for Exp_6 as well as Exp_7. The integrated data includes genomic information for 13,285 genotypes distributed across nine single nucleotide polymorphism (SNP) arrays, based on the Illumina Infinium array [49], with varying marker densities and overlap (Additional file 1: Table S11). A reference chip (chip 9) was selected for genomic data integration since it had the maximum marker overlap with the other SNP arrays. The marker sequences [49] were used to determine the marker physical position [50] (*Triticum aestivum* release-51) using BLAST v2.12.0 [51]. A custom pipeline was used to integrate the SNP arrays [52–54] (Additional file 3: Material S8).

The integrated genomic data were filtered for markers with missing values (threshold = 0.5) and converted to a VCF format [55] for haplotype-based imputation using Beagle v5.2 [56]. Downstream processing involved the use of vcftools v0.1.16 [55] and plink v1.9 [57] to convert the VCF data into numeric data coded 0, 1, 2. After imputation, 10,186 SNPs remained for all lines. Filtering for monomorphic markers and minor allele frequency of 0.01 finally resulted in 9,797 high-quality SNP markers. The SNP profiles of the hybrids were then derived from the genomic data of the parents. The imputation procedure applied here has been shown to yield high accuracies in integrated datasets of comparable structure, including those sharing line material with this study [37].

The F_st_ statistic [24, 25] was calculated segment-wise with a marker window size of 299,999 (~ 0.3 Mb) and a window step size of 29,999. A weighted estimate was then derived for pairwise comparisons of Exp_1 to 7 and a distance matrix was populated. Groupings were analyzed using hierarchical cluster analysis, using *hclust* function [48], and visualized using dendextend v1.17 [58].

From the integrated genomic data, relationship matrices for additive (${GRM}_{a}$), additive epistatic (${GRM}_{aa}$) [4], and dominance (${GRM}_{d}$) [59] effects were calculated (see Additional file 3: Material S9 for more details).

### Environment data

Climate data describing seasonal weather conditions were available at daily resolution [60]. These were used to derive environment variables (EVs) to account for variations between different environments. EVs were derived for each site-year combination and calculated as monthly mean values of 27 parameters (listed in Additional file 1: Table S5) starting 1^st^ October of the sowing year and ending 31^st^ August of the harvest year. The environment relationship matrix was then calculated as:

3 $${ERM}_{l}=\frac{EV*{EV}^{T}}{mean\left(diagonal\left(EV*{EV}^{T}\right)\right)}$$

4 $${ERM}_{nl}=\frac{exp\left(\frac{dist\left(EV\right)}{\theta }\right)}{mean\left(diagonal\left(exp\left(\frac{dist\left(EV\right)}{\theta }\right)\right)\right)}$$

where $EV$ is the scaled environment variable(s) matrix, and ${EV}^{T}$ is transpose of the $EV$ matrix, *dist(EV)* is the Euclidean distance matrix of $EV$, and θ is a scaling factor. The ${ERM}_{l}$ is a proxy for the kinship between respective environments. The ${ERM}_{nl}$ matrix builds upon it and represents a Gaussian kernel for environmental effects [61]. In addition, a year relationship matrix ($YRM$) and site relationship matrix ($SRM$) were similarly derived [as (3)] by characterizing years with mean monthly parameter values across sites and characterizing sites with mean monthly parameter values across years. Hierarchical cluster analysis was performed and the resulting dendrograms were visualized using the same R packages mentioned in the previous section [48, 58].

For 54 out of the total (117) environments, crop management as well as soil data were also added. Management data were scraped from breeders as well as company records. The maximum information received for a trial in a given environment consisted of site GPS (geographic coordinate system) information, altitude (m), sowing date, sowing rate (seeds/m2), harvest date and harvest net area (m2). The minimum information received were site GPS coordinates and sowing date. The missing information was approximated based on domain-specific knowledge, experience of the breeder responsible for the trial and past trial trends at a given site. Lastly, for soil data, maximum rooting depth, thickness of soil layer, soil organic carbon, soil texture by KA5 texture class [62], (including soil sand and clay content), bulk density, soil pH, and initial soil nitrogen for each site were derived from the Bodenübersichtskarte (BÜK) 200 dataset, developed by the Federal Institute for Geosciences and Natural Resources (BGR) [63]. This dataset is originally based on a 1:200,000 scale [64] and has been transformed into 1 km × 1 km grids for large-scale modeling purposes. The soil properties of the experimental sites within the corresponding 1 km grid were used.

### Genotype growth parametrization

Crop growth model MONICA is a process-based simulation model for nitrogen and carbon dynamics in agroecosystems, evolved from the HERMES model [21]. It incorporates representations of soil–plant–atmosphere interactions, accounting for processes such as evapotranspiration, soil moisture movement, nitrogen mineralization, abiotic stresses (including heat, drought, nitrogen, and waterlogging), and plant nutrient uptake. In this study, it was used to parameterize genotype growth across study sites in two sequential steps: (1) parameterizing genotype phenology at each site, and (2) using step 1 results to parameterize biomass accumulation and yield formation. In both steps, we used the sequential uncertainty fitting algorithm (SUFI-2) technique [65] for model calibration–a process which searches for optimal model parameters to minimize prediction uncertainty on observed data. SUFI-2 maps all model uncertainties onto parameter ranges, with uncertainty quantified by 95% prediction uncertainty calculated at the 2.5% and 97.5% levels of cumulative distribution using Latin Hypercube sampling. This method accounts for both parameter sensitivity and interactions, enabling robust parameter estimation even under limited or noisy observed data. A total of 200 parameter combinations were generated at each calibration step.

In the first step, the model was calibrated for phenology related parameters, including base temperatures, day length and vernalization correction factors, and temperature sums required for various growth stages. The specific growth stages [66] were defined as follows: sowing to emergence (BBCH 9), emergence to double ridge (BBCH 30–31), double ridge to heading (BBCH 55) (Additional file 3: Material S2), heading to anthesis (BBCH 65), and anthesis to maturity (BBCH 89), representing key developmental milestones in cereal crops. The primary reference variables used to calibrate crop phenology were the heading and harvest dates. The harvest date is not a true phenological stage, however due to the absence of available maturity dates, harvest dates were assumed to serve as proxies for maturity. The observed and simulated heading and harvest dates were compared for all 200 parameter sets using mean absolute error function. The parameter set with minimum error was selected for the second step of calibration, where parameters describing biomass accumulation and yield formation were derived for each growth stage. The climate, soil, and management information as well as observed yield data were used for this purpose. Additional file 1: Table S7 shows the list of parameters with their maximum and minimum values used during model calibration.

### Model frameworks

Genome-wide predictions were considered for within-environment means, but also for across-environment means i.e. BLUEs from model (1). See Fig. 6 for complete workflow overview. For the latter, the standard GBLUP model [4] based on additive effects was extended stepwise as,

GBLUP_D $$y\;=\;\mu+G_a\;+G_d\;+e$$

E-GBLUP_D $$y\;=\;\mu\;+\;G_a\;+\;G_d\;+\;G_{aa}\;+\;e$$

Where $y$ is a vector of phenotypic values, ${G}_{a}\sim N(0, {GRM}_{a}{\sigma }_{a}^{2})$*,* ${G}_{d}\sim N\left(0, {GRM}_{d}{\sigma }_{d}^{2}\right)$ and ${G}_{aa}\sim N(0, {GRM}_{aa}{\sigma }_{aa}^{2})$ are vectors of additive, dominance and additive epistatic genotypic effects respectively. $e \sim N(0, {I}_{e}{\sigma }_{e}^{2})$ is the model residual. ${I}_{e}$ is the corresponding identity matrix. The covariance structures for respective genotypic effects (${GRM}_{a, d, aa}$) were defined previously (Additional file 3: Material S9). Model parameters (${\sigma }_{a}^{2}$, ${\sigma }_{d}^{2}$, ${\sigma }_{aa}^{2}$, and ${\sigma }_{e}^{2}$) were estimated and predictions were done using mixed linear (Bayesian regression) models with BGLR framework [67] with parameters “*nIter*” and “*burnIn*” set to 15,000 and 2,000 respectively.

Models GBLUP_D and E-GBLUP_D were compared to convolutional neural network model, acr_CNN, regressing additive marker effects on grain yield phenotypic values. The acr_CNN was designed as a hyperparameter tuner with “keras” framework [68]. For each prediction task, the tuner object optimized the acr_CNN architecture within bounds specified as hyperparameter space–this is called hyperparameter tuning. The general structure of the tuner included a fixed one-dimensional convolution input + average pooling layer, followed by variable number of convolutions + average pooling layers leading into flattening layer. This was followed by variable number of densely connected + dropout layers and a fixed output layer (more details at Additional file 3: Material S1). The convolution + pooling part was used for hierarchical feature extraction from genomic data, with successive layers capturing increasingly complex feature representations and reducing dimensionality through pooling. The dense + dropout part was used for learning complex non-linear relationships between the extracted features and phenotypic data while preventing overfitting through dropout regularization.

For genome-wide predictions of means within environments, i.e. phenotypic data only corrected for the statistical effects of the field trial designs, the standard GBLUP model was extended stepwise and the model M_1 to M_8 were implemented as:

M_1 $$y ={\upmu +{Z}_{E} E}_{I} + {{Z}_{G} G}_{I} + e$$

Where, *y* is vector of phenotypic values ordered as genotypes within environments. ${E}_{I}\sim N(0, {I}_{E}{\sigma }_{E}^{2})$ is the vector of main environment effects, ${G}_{I}\sim N(0, {I}_{G}{\sigma }_{G}^{2})$ is the vector of main genotype effects and $e \sim N(0, {I}_{e}{\sigma }_{e}^{2})$ is the model residual. ${Z}_{E}$ and ${Z}_{G}$ are the design matrices for corresponding effects. ${I}_{E}$, ${I}_{G}$ and ${I}_{e}$ are the identity matrices used to model the uncorrelated random effects of environments, genotypes and residuals, respectively.

M_2 $$y={\upmu +{Z}_{E} E}_{I}+{{Z}_{G}(G}_{a}+{G}_{d}+{G}_{aa})+e$$

Common terms have similar meaning to M_1, except ${G}_{a}\sim N(0, {GRM}_{a}{\sigma }_{a}^{2})$*,* ${G}_{d}\sim N\left(0, {GRM}_{d}{\sigma }_{d}^{2}\right)$ and ${G}_{aa}\sim N(0, {GRM}_{aa}{\sigma }_{aa}^{2})$ are vectors of additive, dominance and additive epistatic genotypic effects. The genomic relationship matrices (${GRM}_{a, d, aa}$, Additional file 3: Material S9) exploit pairwise genotype relationships when predicting respective effects.

M_3 $$y={\upmu +{Z}_{E} E}_{l}+{{Z}_{G}(G}_{a}+{G}_{d}+{G}_{aa})+e$$

Common terms have similar meaning to models before, except ${E}_{l}\sim N(0, {ERM}_{l}{\sigma }_{l}^{2})$ is the vector of environment effects. The genomic relationship matrices ${ERM}_{l}$ were defined as in formula (3) and capture pairwise environment relationships when predicting respective effects.

M_4 $$y={\upmu +{Z}_{E} E}_{l}+{{Z}_{G}(G}_{a}+{G}_{d}+{G}_{aa})+{GE}_{1}+e$$

Common terms have similar meaning to models before, except ${GE}_{1}\sim N(0, {GERM}_{1}{\sigma }_{{GE}_{1}}^{2})$ is the vector of genotype-times-environment effects, with ${GERM}_{1}={({Z}_{E}ERM}_{l}{Z}_{E}^{T})\odot {({Z}_{G}GRM}_{a}{Z}_{G}^{T})$. “$\odot$” represents a Hadamard (element-wise) product and superscript “T” denotes transpose of the matrix.

M_5 $$y={\upmu +{Z}_{E} E}_{nl}+{{Z}_{G}(G}_{a}+{G}_{d}+{G}_{aa})+e$$

Common terms have similar meaning to models before, except ${E}_{nl}\sim N(0, {ERM}_{nl}{\sigma }_{nl}^{2})$ is the vector of environment effects. The environment relationship matrices ${ERM}_{nl}$ were defined as in formula (4) and captures pairwise, potentially non-linear, environment relationships when predicting respective effects.

M_6 $$y={\upmu +{Z}_{E} E}_{nl}+{{Z}_{G}(G}_{a}+{G}_{d}+{G}_{aa})+{GE}_{2}+e$$

Common terms have similar meaning to models before, except ${{GE}}_{2}\sim \text{ N}(0, {{GERM}}_{2}{\upsigma }_{{{GE}}_{2}}^{2})$ is the vector of genotype times environment effects, with ${{GERM}}_{2}=({{{Z}}_{{E}}{ERM}}_{{nl}}{{Z}}_{{E}}^{{T}})\odot ({{{Z}}_{{G}}{GRM}}_{{a}}{{Z}}_{{G}}^{{T}})$. This is an analogue of M_4, enhancing modeling of higher order genotype times environment interactions.

M_7 $$y={\upmu +Z}_{S}S+{Z}_{Y}Y+{Z}_{G}\left({G}_{a}+{G}_{d}\right)+{GY}_{a}+{Z}_{GS}G{S}_{I}+{GE}_{1}+e$$

Common terms have similar meaning to models before, except $S \sim N(0, SRM{\sigma }_{s}^{2})$ is a vector of site effects, $Y \sim N(0, YRM{\sigma }_{Y}^{2})$ is a vector of year effects–$YRM$ and $SRM$ were defined previously. ${GY}_{a}\sim N(0, {GYRM}_{a}{\sigma }_{{GY}_{a}}^{2})$ is a vector of genotype-year interaction effects–with ${{GYRM}_{a}=({Z}_{Y}YRM{Z}_{Y}^{T})\odot ({Z}_{G}GRM}_{a}{Z}_{G}^{T})$. Lastly, $G{S}_{I}\sim N(0, {I}_{{GS}_{I}}{\sigma }_{{GS}_{I}}^{2})$ is the vector of main genotype-site effects. ${Z}_{S}$, ${Z}_{Y}$, ${Z}_{GS}$ are the corresponding design matrices for the effects and ${I}_{{GS}_{I}}$ is the respective diagonal identity matrix.

M_8 $$y={\upmu +Z}_{S}S+{Z}_{Y}Y+{Z}_{G}\left({G}_{a}+{G}_{d}\right)+{GY}_{a}+GS+{GE}_{1}+e$$

Common terms have similar meaning to models before, except $GS\sim N(0, {I}_{GS}{\sigma }_{GS}^{2})$ is the vector of main genotype-site effects and modelled with outputs as derived from crop growth model MONICA (Additional file 1: Table S7). ${I}_{GS}$ is the identity matrix. All model variances (${\sigma }_{x}^{2}$, where $x\in \{e, g, a, d, aa, l, nl,{ GE}_{1},{ GE}_{2}, S, Y, G{S}_{I}, GS\}$) were estimated and predictions were done using mixed linear (Bayesian regression) models with BGLR framework [67] with parameters “*nIter*” and “*burnIn*” set to 15,000 and 2,000 respectively.

Models M_1 to M_8 were compared to two convolutional neural network based models, CNN_EV and CNN_GS. CNN_EV consisted of two separate convolutional branches: one for additive marker effects to model genotypic influences, and another for environmental variables to capture environmental effects. The former had three convolutional + average pooling layers, while the latter had three convolutional layers without average pooling layers (more details at Additional file 3: Material S4). These were sequentially concatenated and later connected to a variable number of densely connected + dropout layers and a fixed output layer. Notably, only the densely connected part was tuned here due to memory constraints with larger model sizes on a single graphic processing unit. CNN_GS shared the same architecture as CNN_EV, with the key difference being that one convolutional branch took genotype-site-specific MONICA-derived parameters in place of environmental variables. The dual-branch design allowed independent processing of input data to capture distinct feature representations before concatenation. The branch processing genomic data used pooling layers to reduce dimensionality while preserving informative features from successive convolutions, whereas the branch processing environmental variables/MONICA-derived parameters omitted pooling to maintain full resolution of respective features. The dense layers were used to capture genotype times environment interactions from the concatenated features and map these to phenotypic data while using dropout regularization to prevent overfitting.

Minimally preprocessed data were used for the CNNs. The input data was scaled between (0, 1) using *MinMaxScaler* function of scikit-learn v1.0.2 [69] and reshaped appropriate to the specific cross validation run with numeric data type set to ‘float32’. The implementation of the CNNs required 20% of the genotypes of the training population to be used for a validation set. The training and validation sets were used to tune the hyperparameters, learning the weights, and biases to produce a learned model for a given for each prediction task.

Model tuning was done using the hyperband optimization algorithm [70] with KerasTuner [68]. “mean_squared_error” validation loss calculated for batches of training data fed into a model with randomly initiated hyperspace within specified bounds (Additional file 3: Materials S1 and 4), was monitored to derive best hyperparameters during the tuning process. Early stopping was implemented to monitor validation loss on the validation set with patience = 5 and min_delta = 0.001. For hyperparameter tuning, epoch, factor and hyperband iterations were set to 100, 4, and 1, respectively. During model fitting, the best model was extracted to initialize the CNN and the training data were used to learn the model weights and biases with batch size of 32 over 100 epochs. In the model fitting, early stopping was implemented with same parameter values as before, except that min_delta was set to 0.00001. The fitted model was used to predict grain yield using the test dataset and the output was scaled back to the original units.

The GBLUP-based models were implemented in R v4.0.5 [48], while the convolutional neural networks were developed with TensorFlow v2.8 [71] and implemented in Python v3.8 [72]. Prediction tasks were converted into jobs for in-house computing clusters and executed within a computing environment [73] based on the NVIDIA runtime image [74], to ensure reproducibility. The clusters were managed using Simple Linux Utility for Resource Management [75], and included both CPU-only and GPU-enabled nodes, all operating on Rocky Linux 9 with x86_64 architecture. The CPU-only nodes had between 28 to 128 cores (56 to 256 threads) and 768 GB to 3 TB of RAM, with some nodes dedicated to interactive or heterogeneous computing. The GPU-enabled nodes had 48 to 128 CPU cores (96 to 256 threads), 1 TB of RAM, and were equipped with various GPUs: 6–7 × NVIDIA A40, or 3 × NVIDIA A100. Care was taken to adjust for R’s 1-based and Python’s 0-based indexing when transferring data, and to ensure compatibility of data types, file encoding, and metadata for smooth interoperability between the two environments. Resources and runtimes used for different models are noted in Additional file 1: Tables S10 and 12, respectively.

### Cross validation scenarios to compare models

To evaluate the performance of a model for predicting mean genotypic values across environments, two cross validation scenarios were investigated: (1) Traditional five-fold cross validation (Fig. 2a). Here, the genotypes were randomly divided into a training and a test population in an 80:20 ratio, treating genomic duplicates as a single distinct genotype. The test set was predicted and results from all folds of a run were stacked to calculate Pearson correlation coefficient (prediction ability). This validation was run 10 times, yielding 50 total model evaluations (10 runs × 5 folds). (2) Scenario based validation (Fig. 2b) with subsampling of increasing numbers of training populations. Six scenarios with gradually increasing training population sizes were implemented–each with varying number of splits. When genomic duplicates appeared across multiple experimental series, their natural distribution was preserved rather than removing all instances except one from each series. Prediction ability in this case was calculated specific to each split.

Four cross validation scenarios were used (Fig. 3a) to predict grain yield of (1) known genotypes in known environments (cv1), (2) known genotypes in new environments (cv2), (3) new genotypes in known environments (cv3) and (4) new genotypes in new environments (cv4). cv1 to cv4 were run 50 times. Additionally, leave-one-environment-out (LoO) cross validation was performed, in which the performance of genotypes in a given environment was predicted based on information from other environments in which they were present. To account for the highly unbalanced nature of the data, it was ensured that the training dataset contained at least three data points for each combination of genotype–environment in the test set, and 38 possible LoO runs were tested.

Prediction ability was calculated for each test set environment, cross validation scenario, and run. Thereafter, mean correlation coefficient was reported for each cross validation scenario and run. Mean correlations for M_1 (as baseline model) were compared to others using a pairwise t-test to test for statistically significant difference with a p-value threshold of 0.05. The predictive abilities of two models were compared by calculating the mean percent difference in performance across cross validation runs.

### Deriving feature importance score for environment variables

A core set of 500 genetically diverse lines was selected with the corehunter package [76] using the genotypic data. Missing grain yield data for these lines were filled using model M_6 across each of the 117 environments (see Fig. 6 for an overview). For this, the grain yield data for all lines–including that of core set–across all environments were used as the training set. Predicted grain yield values for the core set were then extracted, and following linear mixed model was fitted to derive genotype times environment (G × E) patterns while accounting for both genotype and environmental effects:

5 $$\hat{y} = \mu + G{\tau} + Eu + e,$$

where, $\widehat{y}$ is the vector of predicted grain yield values from M_6 ordered as genotypes in respective environments. Rest of the terms have the same meaning as model (1). For extracting residuals from fitted model, *τ*, *u* and *e* were assumed to follow independent, normally distributed random effects. The model was implemented with ASREML-4.2 inside R v4.0.5 [48].

Residuals from this model were used to construct a rectangular matrix representing G × E patterns. Euclidean distances among environments were derived from this matrix, using *dist* function [48]. The K-Means clustering, using *kmeans* function, [27, 48] was then applied to the first two principal coordinates (using *cmdscale* function [48]) derived from this distance matrix to identify environmental clusters. The optimal number of clusters was determined by analyzing two metrics as cluster number increased: (1) within-cluster sum of squared errors and (2) silhouette scores. To maintain adequate sample sizes for training, clusters were required to contain at least three environments each. Additionally, hierarchical clustering (using *hclust* function [48]) was employed to compare tree topologies [58] for environment clustering derived from G × E patterns with those obtained from environment variables (EV). Lastly, cluster information was combined with EVs to derive feature importance scores.

Initially, to evaluate model performance, a gradient boosting classifier [28] was employed with parameters set to learning_rate = 0.1, n_estimators = 3000, and max_depth = 30. For this a validation study was used in which the data were divided into training (80%) and test (20%) sets, sampled randomly. These sets were generated with the *RepeatedStratifiedKFold* function [69], with parameters n_splits = 5, n_repeats = 3, and random_state = 1, ensuring that each environment was included in a test set exactly once per repeat. Accuracy scores were then calculated for each split as a percentage of correctly predicted clusters for environments in the test set. Mean score across 15 train/test (5*3) splits was reported.

Thereafter, the data were divided into training (80%) and validation (20%) sets using the *train_test_split* function [69]. A gradient boosting classifier [28], with parameters as derived previously, was then trained on the training data to derive "Gain"-based importance scores. Both training and validation sets were used to monitor training progress, and early stopping was implemented by setting early_stopping_rounds = 100 with eval_metric = 'mlogloss'. The final "Gain" scores were used to rank the environmental variables.

### Yield gain with enviromically adapted genotypes against those selected based on general performance

Genotypes were ranked using the best linear unbiased estimates of grain yield performance, as derived using model (1) (see Fig. 6 for an overview). Two sets of genotypes were selected: (a) the top 7 percent (subset 1), and (b) a random 3 percent from the remaining genotypes (subset 2). Their performance in all environments was predicted using model M_6 using information of all line genotypes as training data.

## Supplementary Information

Additional file 1. Contains additional Tables S1-12.

Additional file 2. Contains additional Figs. S1-10.

Additional file 3. Contains additional Materials S1-9.

## Data Availability

The data can be accessed as follows: phenotypic data for Exp_1 are provided as supplementary materials in a study published in PNAS [77]; preprocessed, pseudonymized data for Exp_2 to Exp_5 are available via the Zenodo repository [78]; all data for Exp_6 can be requested from KWS LOCHOW GmbH, subject to scientific review and a completed material transfer agreement; and phenotypic and genotypic data for Exp_7 have been published at e!DAL-PGP [47] and Dryad [79]. All other data—including curated genotypic, phenotypic, climate, management, and soil data—are currently being transferred to a data trusteeship platform (http://dtp.ipk-gatersleben.de) developed as part of the DRIVE project (FKZ: 031B1537A) to generate long-term FAIR access to the data. Example code for genomic predictions is available, licensed under the MIT license [80, 81].
