# Supplementary material for: Predicting enviromically adapted varieties with big data
Source: Genome Biol. 2026 Jan 7;27:3. doi: 10.1186/s13059-025-03914-x (PMC12838137; doi:10.1186/s13059-025-03914-x)
Supplement: Supplementary file 1 — Additional file 1. Contains additional Tables S1-12. [file 13059_2025_3914_MOESM1_ESM.docx]

Table S1: Overview of phenotypic data.

| Description | Exp-1 | Exp-2 | Exp-3 | Exp-4 | Exp-5 | Exp-6 | Exp-7 |
| --- | --- | --- | --- | --- | --- | --- | --- |
| Environments | 11 | 12 | 6 | 23 | 15 | 42 | 8 |
| Year (Site + Trial) | 2012 (5), 2013(6) | 2016 (6), 2017 (6) | 2018 (6) | Series 1: 2016 (6), 2017 (5), Series 2: 2017 (6), 2018 (6) | Series 1: 2016 (5), Series 2: 2017 (5), Series 3: 2018 (5) | 2013 (8), 2014 (8), 2015 (9), 2020 (9), 2021 (8) | 2009 (3), 2010 (5) |
| Environments with extended data** |  | 9 | 4 | 15 |  | 24 | 2 |
| Progeny type | Single-cross hybrid progenies | Single-cross hybrid progenies | Single-cross hybrid progenies | Single-cross hybrid progenies | Elite lines x Historic varieties/accessions | Elite cencvMainl European winter wheat lines | European cultivars from 1975 to 2007 |
| Mating design | Factorial | Factorial | Factorial | Factorial | Factorial and Top cross |  |  |
| Males | 15 | 41 | 40 | *Series 1: 57 Series 2: 60 | *Series 1: 107 Series 2: 206 Series 3: 121 |  |  |
| Females | 120 | 188 | 194 | Series 1: 4 Series 2: 4 | Series 1: 148 Series 2: 116  Series 3: 99 |  |  |
| Hybrids | 1604 | 1747 | 1730 | Series 1: 228 Series 2: 240 | Series 1: 337 Series 2: 459 Series 3: 165-276 |  |  |
| Checks | 7 | 11 | 11 | Series 1: 16 Series 2: 16 | Series 1: 18 Series 2: 24-25 Series 3: 24 |  |  |
| Lines | - | - | - | - | - | 5237 | 372 |
| Experimental Design | Partially replicated alpha lattice design | Unreplicated alpha lattice design | Unreplicated alpha lattice design | Unreplicated alpha lattice design | Unreplicated alpha lattice design | Alpha lattice design (1 to 3 replications per site) | Alpha lattice design with two replications per site |
| Plot Sizes | 5 to 7.4 m^2^ | 5.70 to 10.00 m^2^ | 5.70 to 9.00 m^2^ | Series 1: 5.7 to 10.50 m^2^, Series 2: Not specified | 6 to 9 m^2^ | 6.05 to 17.25 m^2^ | 5 to 6.8 m^2^ |
| Grain yield per environment repeatability | Min = 0.27  Median = 0.64  Max = 0.87 |  |  |  |  | Min = 0.55  Median = 0.81  Max = 0.93 | Min = 0.73  Median = 0.87  Max = 0.96 |
| Heading date per environment repeatability |  |  |  |  |  | Min = 0.78  Median = 0.89  Max = 0.99 | Min = 0.94  Median = 0.97  Max = 0.99 |

*Series represents a pool of parents used for hybrid production in a given year. Repeatability values for Exp-1 and 6 were derived from [1] and those of Exp-7 were derived from [2]. ** Extended data refers to crop management and soil data.

Table S2: Genomic repeatabilities for grain yield.

| Series | CvMainit | Min | Median | Mean | Max | Environments |
| --- | --- | --- | --- | --- | --- | --- |
| Exp_1 | grain yield | 0.111 | 0.345 | 0.348 | 0.741 | 11 |
| Exp_2 | grain yield | 0.139 | 0.426 | 0.413 | 0.621 | 12 |
| Exp_3 | grain yield | 0.182 | 0.423 | 0.407 | 0.639 | 6 |
| Exp_4 | grain yield | 0.141 | 0.354 | 0.354 | 0.565 | 23 |
| Exp_5 | grain yield | 0.0682 | 0.453 | 0.410 | 0.583 | 15 |
| Exp_6 | grain yield | 0.240 | 0.397 | 0.406 | 0.642 | 42 |
| Exp_7 | grain yield | 0.305 | 0.467 | 0.441 | 0.529 | 8 |

*Genomic repeatability was calculated as the ratio of genomic variance to the total variance (genomic plus error variance), estimated using the GBLUP model [3].

Table S3: Pairwise genotype overlap between experimental series.

|  | Exp_1 | Exp_2 | Exp_3 | Exp_4 | Exp_5 | Exp_6 | Exp_7 |
| --- | --- | --- | --- | --- | --- | --- | --- |
| Exp_1 |  | 4 | 4 | 5 | 7 | 5 | 4 |
| Exp_2 | 17 |  | 15 | 11 | 11 | 8 | 2 |
| Exp_3 | 9 | 22 |  | 36 | 11 | 8 | 2 |
| Exp_4 | 7 | 14 | 37 |  | 16 | 10 | 2 |
| Exp_5 | 16 | 24 | 19 | 17 |  | 25 | 26 |
| Exp_6 | 8 | 21 | 25 | 11 | 31 |  | 13 |
| Exp_7 | 11 | 5 | 5 | 2 | 27 | 12 |  |

* Lower half of the Table S(colored green) shows the series to series overlap after genomic deduplication, while upper half (colored blue) shows the overlap before genomic deduplication

Table S4: Variance decomposition results with model (2) for derivation of mean grain yield (Quintal per Hectare) across environments.

| Source | Value |
| --- | --- |
| 𝜎^2^*_𝐻𝑦𝑏𝑟𝑖𝑑𝑠_* |  |
| 𝜎^2^*_GCA-Female_* | 8.9^*^ |
| 𝜎^2^*_GCA-Male_* | 3.3^*^ |
| 𝜎^2^*_SCA_* | 2.2^*^ |
| 𝜎^2^*_Lines_* | 29.5^*^ |
| 𝜎^2^*_𝐻𝑦𝑏𝑟𝑖𝑑𝑠*Env_* |  |
| 𝜎^2^*_GCA-Female*Env_* | 4.2^*^ |
| 𝜎^2^*_GCA-Male*Env_* | 2.03^*^ |
| 𝜎^2^*_SCA*Env_* | - |
| 𝜎^2^*_Lines*Env_* | 9.8^*^ |
| 𝜎^2^*_e_* | 19.8 |
| Average No. of environments*_Hybrids_* | 9.15 |
| Average No. of environments*_Lines_* | 5.9 |
| Broad-sense heritability*_Hybrids_* | 0.87 |
| Broad-sense heritability*_Lines_* | 0.89 |

“*” represents the p-value for a likelihood ratio test comparing full model to the reduced model for respective variance components was < 0.05.

Table S5: Overview of climate data.

| Number | Parameter | Short name | Unit | Description |
| --- | --- | --- | --- | --- |
| 1 | air_temp_5cm_avg | at_5_avC | Celsius | Average daily air temperature at five centimeters above ground level. |
| 2 | air_temp_5cm_max | at_5_maC | Celsius | Maximum daily air temperature at five centimeters above ground level. |
| 3 | air_temp_5cm_min | at_5_miC | Celsius | Minimum daily air temperature at five centimeters above ground level. |
| 4 | air_temp_avg | at_avC | Celsius | Average daily air temperature at two meters above ground level. |
| 5 | air_temp_max | at_maC | Celsius | Maximum daily air temperature at two meters above ground level. |
| 6 | air_temp_min | at_miC | Celsius | Minimum daily air temperature at two meters above ground level. |
| 7 | dew_point_avg | dp_avC | Celsius | Average dew point temperature data. |
| 8 | dew_point_max | dp_maC | Celsius | Maximum dew point temperature data. |
| 9 | dew_point_min | dp_miC | Celsius | Minimum dew point temperature data. |
| 10 | pet_period | pre_MM | millimeter | Reference evapocvMainnspiration, calculated using the FAO-56 grass formulation. |
| 11 | precip_acc_period | pre_a_MM | millimeter | Best estimate of liquid-equivalent precipitation. |
| 12 | precip_acc_period_adjusted | pre_ad_MM | millimeter | Amount of liquid-equivalent precipitation, fundamentally derived from the raw precipitation product, but then adjusted to more closely match available ground truth observations. |
| 13 | precip_acc_period_raw | pre_r_MM | millimeter | Amount of liquid-equivalent precipitation, estimated from multiple sources of data that may include any or all of the following: weather radar, satellite, computer model, and surface observation data. |
| 14 | relative_humidity_avg | rh_avP | - | Average relative humidity data. |
| 15 | relative_humidity_max | rh_maP | - | Maximum relative humidity data. |
| 16 | relative_humidity_min | rh_miP | - | Minimum relative humidity data. |
| 17 | long_wave_radiation_avg | lwr_avW | Watt per square meter | Average downwelling longwave radiation flux data. |
| 18 | long_wave_radiation_max | lwr_maW | Watt per square meter | Maximum downwelling longwave radiation flux data. |
| 19 | long_wave_radiation_min | lwr_miW | Watt per square meter | Minimum downwelling longwave radiation flux data. |
| 20 | short_wave_radiation_avg | swr_avW | Watt per square meter | Average downwelling shortwave radiation flux data. |
| 21 | short_wave_radiation_max | swr_maW | Watt per square meter | Maximum downwelling shortwave radiation flux data. |
| 22 | wind_speed_2m_avg | ws_2_avK | Kilometer per hour | Average daily wind speed at two meters above ground level. |
| 23 | wind_speed_2m_max | ws_2_mxK | Kilometer per hour | Maximum daily wind speed at two meters above ground level. |
| 24 | wind_speed_2m_min | ws_2_miK | Kilometer per hour | Minimum daily wind speed at two meters above ground level. |
| 25 | wind_speed_avg | ws_avK | Kilometer per hour | Average daily wind speed at ten meters above ground level. |
| 26 | wind_speed_max | ws_maK | Kilometer per hour | Maximum daily wind speed at ten meters above ground level. |
| 27 | wind_speed_min | ws_miK | Kilometer per hour | Minimum daily wind speed at ten meters above ground level. |

Additional information at <https://docs.clearag.com/documentation/Weather_Data/Historical_and_Climatological_Weather/latest#_response_object_json_5>

Table S6: The scenarios tested to determine genomic prediction ability.

| Group | scenario | total splits | CNN better* | CNN competitive** | CNN performance*** |
| --- | --- | --- | --- | --- | --- |
| Line | 1 | 7 | 1 | 0 | 0.14 |
| Line | 2 | 21 | 2 | 2 | 0.19 |
| Line | 3 | 35 | 4 | 8 | 0.34 |
| Line | 4 | 35 | 5 | 9 | 0.4 |
| Line | 5 | 21 | 6 | 8 | 0.67 |
| Line | 6 | 7 | 4 | 2 | 0.86 |
| Hybrid | 1 | 7 | 1 | 2 | 0.43 |
| Hybrid | 2 | 21 | 2 | 4 | 0.29 |
| Hybrid | 3 | 35 | 6 | 5 | 0.31 |
| Hybrid | 4 | 35 | 9 | 7 | 0.46 |
| Hybrid | 5 | 20 | 3 | 3 | 0.3 |
| Hybrid | 6 | 5 | 0 | 1 | 0.2 |

* splits where acr_CNN had higher prediction ability value than E-GBLUP_D.

** additional splits where acr_CNN performance was at most 0.06 lower than E-GBLUP_D.

*** expressed as (CNN better + CNN competitive) / total splits.

Table S7: Description of the model parameters output by crop growth model MONICA.

| Process | Parameter name | Lower range | Upper range | Organ | Development stage | Description |
| --- | --- | --- | --- | --- | --- | --- |
| Assimilate partitioning | APC_1 | 0,45 | 0,55 | Root | Emergence | Carbon partitioning to particular organ at specific development stage |
| Assimilate partitioning | APC_10 | 0,2 | 0,23 | leaf | Heading | Carbon partitioning to particular organ at specific development stage |
| Assimilate partitioning | APC_11 | 0,55 | 0,6 | shoot | Heading | Carbon partitioning to particular organ at specific development stage |
| Assimilate partitioning | APC_13 | 0,07 | 0,05 | Root | Anthesis | Carbon partitioning to particular organ at specific development stage |
| Assimilate partitioning | APC_14 | 0,07 | 0,07 | leaf | Anthesis | Carbon partitioning to particular organ at specific development stage |
| Assimilate partitioning | APC_15 | 0,04 | 0,08 | shoot | Anthesis | Carbon partitioning to particular organ at specific development stage |
| Assimilate partitioning | APC_5 | 0,15 | 0,25 | Root | Double ridge | Carbon partitioning to particular organ at specific development stage |
| Assimilate partitioning | APC_6 | 0,15 | 0,25 | leaf | Double ridge | Carbon partitioning to particular organ at specific development stage |
| Assimilate partitioning | APC_9 | 0,07 | 0,12 | Root | Heading | Carbon partitioning to particular organ at specific development stage |
| Phenology & Growth | BaseT_1 | 0 | 5 | - | Emergence | Base temperature for specific development stage |
| Phenology & Growth | BaseT_2 | 0 | 10 | - | Double ridge | Base temperature for specific development stage |
| Phenology & Growth | BaseT_3 | 0 | 10 | - | Heading | Base temperature for specific development stage |
| Phenology & Growth | BaseT_4 | 0 | 10 | - | Anthesis | Base temperature for specific development stage |
| Phenology & Growth | BaseT_5 | 0 | 20 | - | Maturity | Base temperature for specific development stage |
| Heat stress | CTT | 30 | 33 | - | - | Heat stress starting temperature threshold |
| Phenology | DL_1 | 8 | 10 | - | Double ridge | Day length correction factor for specific development stage |
| Phenology | DL_2 | 8 | 10 | - | Heading | Day length correction factor for specific development stage |
| Phenology | DL_3 | 8 | 9 | - | Anthesis | Day length correction factor for specific development stage |
| Drought stress | DST_1 | 0,85 | 0,95 | - | Emergence | Drought stress threshold indicating the sensitivity to drought for specific development stage |
| Drought stress | DST_2 | 0,85 | 0,95 | - | Double ridge | Drought stress threshold indicating the sensitivity to drought for specific development stage |
| Drought stress | DST_3 | 0,85 | 0,95 | - | Heading | Drought stress threshold indicating the sensitivity to drought for specific development stage |
| Drought stress | DST_4 | 0,85 | 0,95 | - | Anthesis | Drought stress threshold indicating the sensitivity to drought for specific development stage |
| Drought stress | DST_5 | 0,85 | 0,95 | - | Maturity | Drought stress threshold indicating the sensitivity to drought for specific development stage |
| Canopy growth | GR_1 | 0,27 | 0,35 | Leaves | - | Specific organ growth respiration |
| Canopy growth | GR_2 | 0,27 | 0,35 | Stem | - | Specific organ growth respiration |
| Canopy growth | GR_3 | 0,27 | 0,35 | Roots | - | Specific organ growth respiration |
| Canopy growth | GR_4 | 0,27 | 0,35 | Storage | - | Specific organ growth respiration |
| Canopy growth | IPB | 40 | 60 | root | Emergence | Initial plant biomass at emergence |
| Root growth | IRD | 0,085 | 0,015 | Roots | Emergence | Initial rooting depth at emergence |
| Drought stress | KC_1 | 0,55 | 0,65 | - | Emergence | Crop coefficient is a dimensionless factor that relates the crop's ETa to ETo for specific development stage |
| Drought stress | KC_2 | 0,75 | 0,85 | - | Double ridge | Crop coefficient is a dimensionless factor that relates the crop's ETa to ETo for specific development stage |
| Drought stress | KC_3 | 0,95 | 1 | - | Heading | Crop coefficient is a dimensionless factor that relates the crop's ETa to ETo for specific development stage |
| Drought stress | KC_4 | 0,95 | 1 | - | Anthesis | Crop coefficient is a dimensionless factor that relates the crop's ETa to ETo for specific development stage |
| Drought stress | KC_5 | 0,65 | 0,75 | - | Maturity | Crop coefficient is a dimensionless factor that relates the crop's ETa to ETo for specific development stage |
| Canopy growth | LSen_1 | 0,03 | 0,05 | Leaves | Anthesis | Leaf senescence rate for specific development stage |
| Canopy growth | LSen_2 | 0,03 | 0,05 | Leaves | Maturity | Leaf senescence rate for specific development stage |
| Canopy growth | MAR | 38 | 52 | - | - | Maximum carbon assimilation rate |
| Canopy growth | MR_1 | 0,01 | 0,02 | Leaves | - | Specific organ maintenance respiration |
| Canopy growth | MR_2 | 0,025 | 0,035 | Stem | - | Specific organ maintenance respiration |
| Canopy growth | MR_3 | 0,01 | 0,02 | Roots | - | Specific organ maintenance respiration |
| Canopy growth | MR_4 | 0,01 | 0,02 | Storage | - | Specific organ maintenance respiration |
| Canopy growth | RF | 0,05 | 0,3 | Storage | Maturity | Carbon remobilization from stem to storage organs at the end of grain filling |
| Root growth | RPR | 0,001 | 0,006 | Roots | - | Root penecvMaintion rate |
| Canopy growth | SLA_1 | 0,001 | 0,0015 | Leaves | Emergence | Specific leaf area for particular development stage |
| Canopy growth | SLA_2 | 0,0019 | 0,0025 | Leaves | Double ridge | Specific leaf area for particular development stage |
| Canopy growth | SLA_3 | 0,0019 | 0,0025 | Leaves | Heading | Specific leaf area for particular development stage |
| Canopy growth | SLA_4 | 0,0018 | 0,0025 | Leaves | Anthesis | Specific leaf area for particular development stage |
| Canopy growth | SLA_5 | 0,0018 | 0,0025 | Leaves | Maturity | Specific leaf area for particular development stage |
| Phenology | Tsum_1 | 50 | 250 | - | Emergence | Temperature sum required to reach specific developmental stage |
| Phenology | Tsum_2 | 400 | 600 | - | Double ridge | Temperature sum required to reach specific developmental stage |
| Phenology | Tsum_3 | 400 | 600 | - | Heading | Temperature sum required to reach specific developmental stage |
| Phenology | Tsum_4 | 350 | 550 | - | Anthesis | Temperature sum required to reach specific developmental stage |
| Phenology | Tsum_5 | 300 | 700 | - | Maturity | Temperature sum required to reach specific developmental stage |
| Phenology | VRN_1 | 0,8 | 1 | - | Double ridge | Vernalization correction factor for specific development stage |
| Phenology | VRN_2 | 0,8 | 1 | - | Heading | Vernalization correction factor for specific development stage |
| Phenology | VRN_3 | 0,8 | 1 | - | Anthesis | Vernalization correction factor for specific development stage |

Table S8: Distribution of 500 core genotypes sampled with corehunter package. Class is the range of environments the core genotypes are present in. Exp_multi represents those genotypes which are present in more than one series.

| Class | Exp_1 | Exp_2 | Exp_3 | Exp_4 | Exp_5 | Exp_6 | Exp_7 | Exp_multi | total |
| --- | --- | --- | --- | --- | --- | --- | --- | --- | --- |
| (1, 3) |  |  |  |  | 1 | 73 |  |  | 74 |
| (4, 6) |  |  | 24 |  | 135 | 119 |  |  | 278 |
| (7, 9) | 7 |  |  | 1 | 1 | 12 | 48 |  | 69 |
| (10, 12) | 10 | 33 |  | 8 | 4 | 9 |  |  | 64 |
| (13, 15) |  |  |  |  | 1 | 4 |  | 2 | 7 |
| (16, 18) |  |  |  |  |  | 1 |  | 3 | 4 |
| (19, 21) |  |  |  |  |  | 1 |  |  | 1 |
| (22, 24) |  |  |  |  |  |  |  | 1 | 1 |
| (49, 51) |  |  |  |  |  |  |  | 1 | 1 |
| (52, 54) |  |  |  |  |  |  |  | 1 | 1 |

Table S9: Mean pairwise overlap of top performers in clusters 1 to 9.

|  | 1 | 2 | 3 | 4 | 5 | 6 | 7 | 8 | 9 |
| --- | --- | --- | --- | --- | --- | --- | --- | --- | --- |
| 1 | 33.4 | 31.4 | 30.2 | 30.2 | 33.9 | 29.2 | 26.4 | 29.3 | 26.8 |
| 2 |  | 30.2 | 28.2 | 28.6 | 30.7 | 26.7 | 27.6 | 27 | 27.3 |
| 3 |  |  | 42.4 | 30.8 | 34.2 | 36.2 | 31 | 37.5 | 19.7 |
| 4 |  |  |  | 30.3 | 30.9 | 30.2 | 29.8 | 29.6 | 24.8 |
| 5 |  |  |  |  | 34.9 | 31.9 | 29.4 | 32.7 | 25.7 |
| 6 |  |  |  |  |  | 42.5 | 30.9 | 32.9 | 20.3 |
| 7 |  |  |  |  |  |  | 39.4 | 31.2 | 23.8 |
| 8 |  |  |  |  |  |  |  | 34.8 | 21.5 |
| 9 |  |  |  |  |  |  |  |  | 51.7 |

* Exceptions with pairwise values greater than respective diagonal values are highlighted in red.

Table S10: Overview of hardware resources used for different model types.

| Model | CPUs (threads) | RAM (GB) | GPUs |
| --- | --- | --- | --- |
| GBLUP_D | 2 | 50 |  |
| E-GBLUP_D | 3 | 50 |  |
| acr_CNN | 1 | 20 | 1 |
| M_1 | 2 | 30 |  |
| M_2 | 4 | 50 |  |
| M_3 | 4 | 100 |  |
| M_4 | 5 | 200 |  |
| M_5 | 4 | 100 |  |
| M_6 | 5 | 200 |  |
| M_7 | 7 | 200 |  |
| M_8 | 7 | 200 |  |
| CNN_EV | 1 | 100 | 1 |
| CNN_GS | 1 | 100 | 1 |

Table S11: Overview of genotypic data derived from nine sources (chip_1 to chip_9). The upper triangular half of the columns 2 to 9 shows the marker overlap between pairs of sources. The tenth and the last column show the absolute number of markers in any given array and experimental series genotypes with it, respectively.

|  | chip_2 | chip_3 | chip_4 | chip_5 | chip_6 | chip_7 | chip_8 | chip_9 | Markers | Experimental series |
| --- | --- | --- | --- | --- | --- | --- | --- | --- | --- | --- |
| chip_1 | 20821 | 14245 | 17835 | 16264 | 13584 | 11264 | 12552 | 17313 | 21062 | 6 |
| chip_2 |  | 14245 | 17594 | 16264 | 13345 | 11264 | 12552 | 17313 | 20821 | 6 |
| chip_3 |  |  | 34394 | 32729 | 11496 | 9769 | 10796 | 35865 | 35865 | 1 |
| chip_4 |  |  |  | 75676 | 14388 | 11380 | 12275 | 77977 | 81489 | 6 |
| chip_5 |  |  |  |  | 13000 | 11157 | 11603 | 75731 | 75761 | 6 |
| chip_6 |  |  |  |  |  | 10814 | 11897 | 13735 | 14411 | 6 |
| chip_7 |  |  |  |  |  |  | 10221 | 11730 | 11736 | 6 |
| chip_8 |  |  |  |  |  |  |  | 12896 | 13006 | 2,3,4,5 |
| chip_9 |  |  |  |  |  |  |  |  | 81587 | 7 |

Table S12: Overview of runtime for different models and cross validation types.

| Validation type | Model | Median runtime | Minimum runtime | Maximum runtime |
| --- | --- | --- | --- | --- |
| 5-Fold | GBLUP_D | 32M | 27M | 1H 11M |
| 5-Fold | E-GBLUP_D | 1H 18M | 44M | 3H 57M |
| 5-Fold | acr_CNN | 3H 13M | 2H 52M | 5H 29M |
| Scenario based | GBLUP_D | 52M | 26M | 1H 50M |
| Scenario based | E-GBLUP_D | 1H 19M | 41M | 3H 43M |
| Scenario based | acr_CNN | 2H 20M | 16M | 6H 6M |
| cv1 | M_1 | 23M | 13M | 1H 22M |
| cv2 | M_1 | 34M | 24M | 2H 1M |
| cv3 | M_1 | 39M | 30M | 2H 10M |
| cv4 | M_1 | 39M | 22M | 2H 5M |
| cv1 | M_2 | 1H 10M | 21M | 2H 48M |
| cv2 | M_2 | 58M | 34M | 3H 2M |
| cv3 | M_2 | 1H 16M | 37M | 3H 5M |
| cv4 | M_2 | 1H 1M | 34M | 3H 8M |
| cv1 | M_3 | 1H 2M | 21M | 3H 0M |
| cv2 | M_3 | 1H 23M | 35M | 3H 9M |
| cv3 | M_3 | 1H 37M | 43M | 4H 40M |
| cv4 | M_3 | 1H 34M | 35M | 3H 14M |
| cv1 | M_4 | 3H 48M | 1H 44M | 7H 28M |
| cv2 | M_4 | 7H 55M | 4H 10M | 18H 35M |
| cv3 | M_4 | 6H 43M | 3H 2M | 11H 4M |
| cv4 | M_4 | 4H 41M | 2H 11M | 7H 8M |
| cv1 | M_5 | 52M | 22M | 3H 11M |
| cv2 | M_5 | 1H 10M | 33M | 3H 24M |
| cv3 | M_5 | 1H 33M | 41M | 4H 0M |
| cv4 | M_5 | 1H 10M | 37M | 4H 16M |
| cv1 | M_6 | 3H 37M | 1H 22M | 8H 32M |
| cv2 | M_6 | 7H 2M | 3H 3M | 14H 14M |
| cv3 | M_6 | 6H 25M | 2H 45M | 10H 8M |
| cv4 | M_6 | 5H 3M | 2H 19M | 7H 39M |
| cv1 | M_7 | 6H 37M | 2H 55M | 19H 14M |
| cv1 | M_8 | 4H 54M | 3M | 9H 23M |
| cv1 | CNN_EV | 5H 45M | 4H 20M | 9H 24M |
| cv2 | CNN_EV | 7H 54M | 4H 10M | 9H 45M |
| cv3 | CNN_EV | 8H 12M | 3H 52M | 9H 48M |
| cv4 | CNN_EV | 8H 0M | 4H 28M | 10H 19M |
| cv1 | CNN_GS | 5H 29M | 4H 41M | 10H 32M |
| LoO | M_1 | 2M | 1M | 21M |
| LoO | M_2 | 5M | 1M | 13M |
| LoO | M_3 | 5M | 1M | 24M |
| LoO | M_4 | 16M | 2M | 1H 6M |
| LoO | M_5 | 5M | 1M | 19M |
| LoO | M_6 | 17M | 2M | 1H 6M |

*Runtimes are expressed in hours (H) and minutes (M) and are rounded to the next minute.

References

1. Zhao Y, Thorwarth P, Jiang Y, Philipp N, Schulthess AW, Gils M, et al. Unlocking big data doubled the accuracy in predicting the grain yield in hybrid wheat. Science Advances. 2021;7:eabf9106. https://doi.org/10.1126/sciadv.abf9106

2. Gogna A, Schulthess AW, Röder MS, Ganal MW, Reif JC. Gabi wheat a panel of European elite lines as central stock for wheat genetic research. Scientific Data. 2022;9:538. https://doi.org/10.1038/s41597-022-01651-5

3. Jiang Y, Reif JC. Modeling epistasis in genomic selection. Genetics. Genetics Soc America; 2015;201:759–68.
