# Supplementary material for: Predicting enviromically adapted varieties with big data
Source: Genome Biol. 2026 Jan 7;27:3. doi: 10.1186/s13059-025-03914-x (PMC12838137; doi:10.1186/s13059-025-03914-x)
Supplement: Supplementary file 2 — Additional file 2. Contains additional Figs. S1-10. [file 13059_2025_3914_MOESM2_ESM.docx]

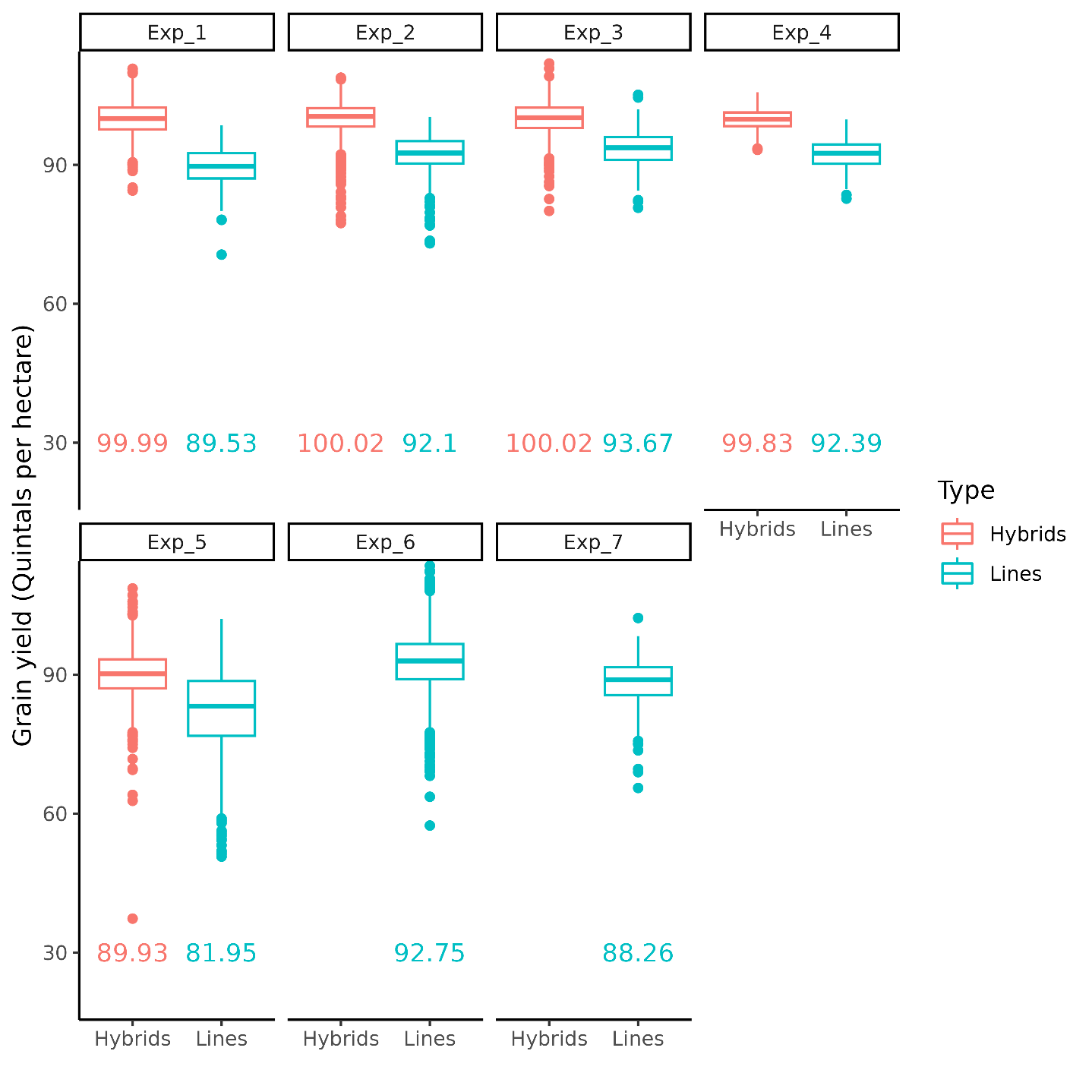


Fig. S1: Grain yield distributions of lines and hybrids in experimental series 1 to 7.


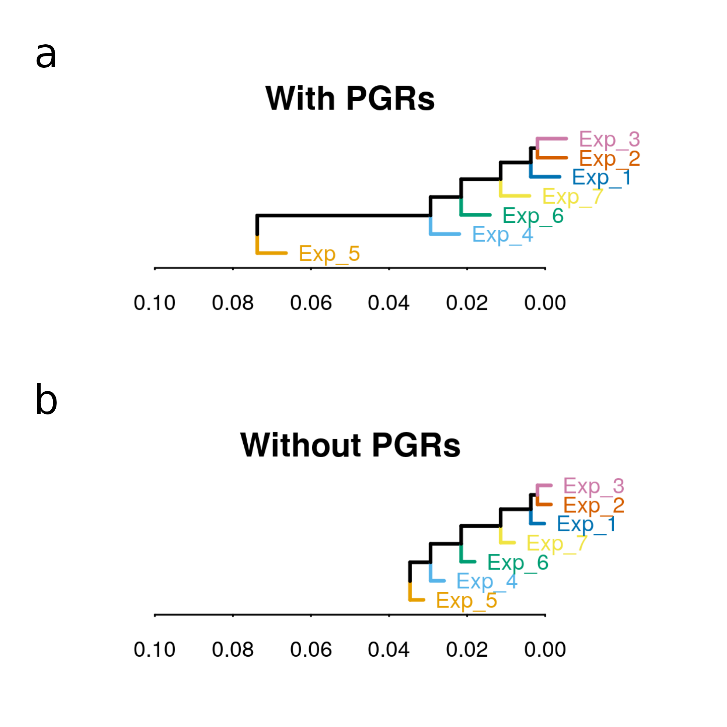


Fig. S2: Population differentiation of major population groups as derived with hierarchical clustering on pairwise F_st_ statistic (on x-axis) between line genotypes (a) with PGR males of Exp_5, (b) without PGR males of Exp_5.


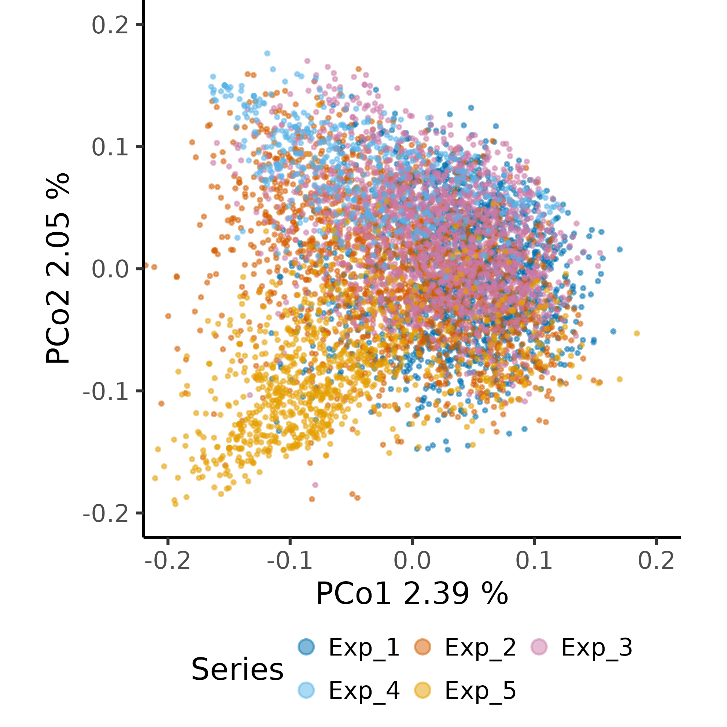


Fig. S3: Genetic diversity space of hybrids with principle coordinate analysis (PCo) plot of Rogers’ distance matrix.


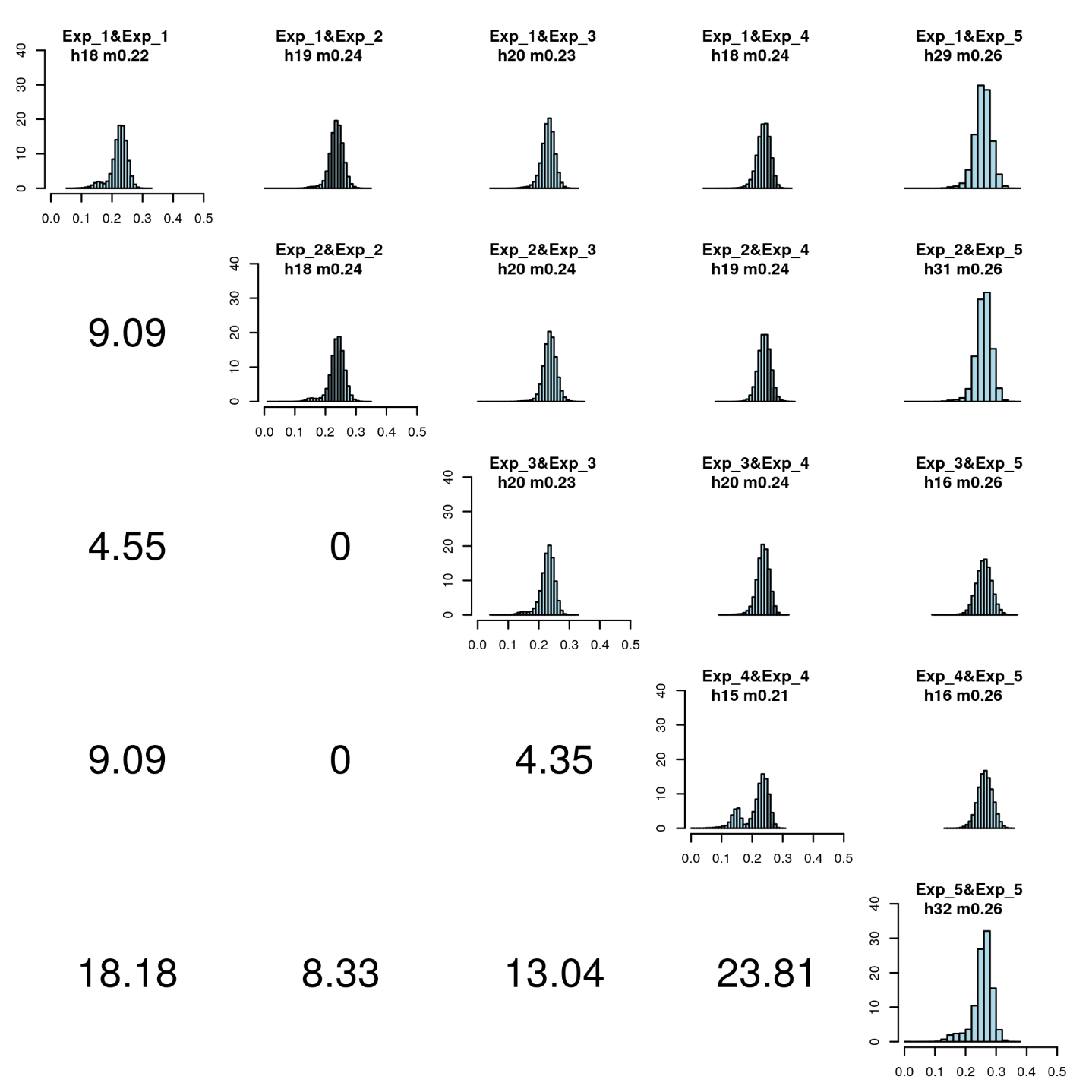


Fig. S4: Distribution of Rogers’ distances for hybrids within and across experimental series. For each histogram the maximum density (y-axis) and distribution mean is reported with “hxx mx.xx” below subFig S title. The off-diagonal values are the percentage changes in mean Rogers’ distance values. Higher values reflect larger genetic distances between the respective series.


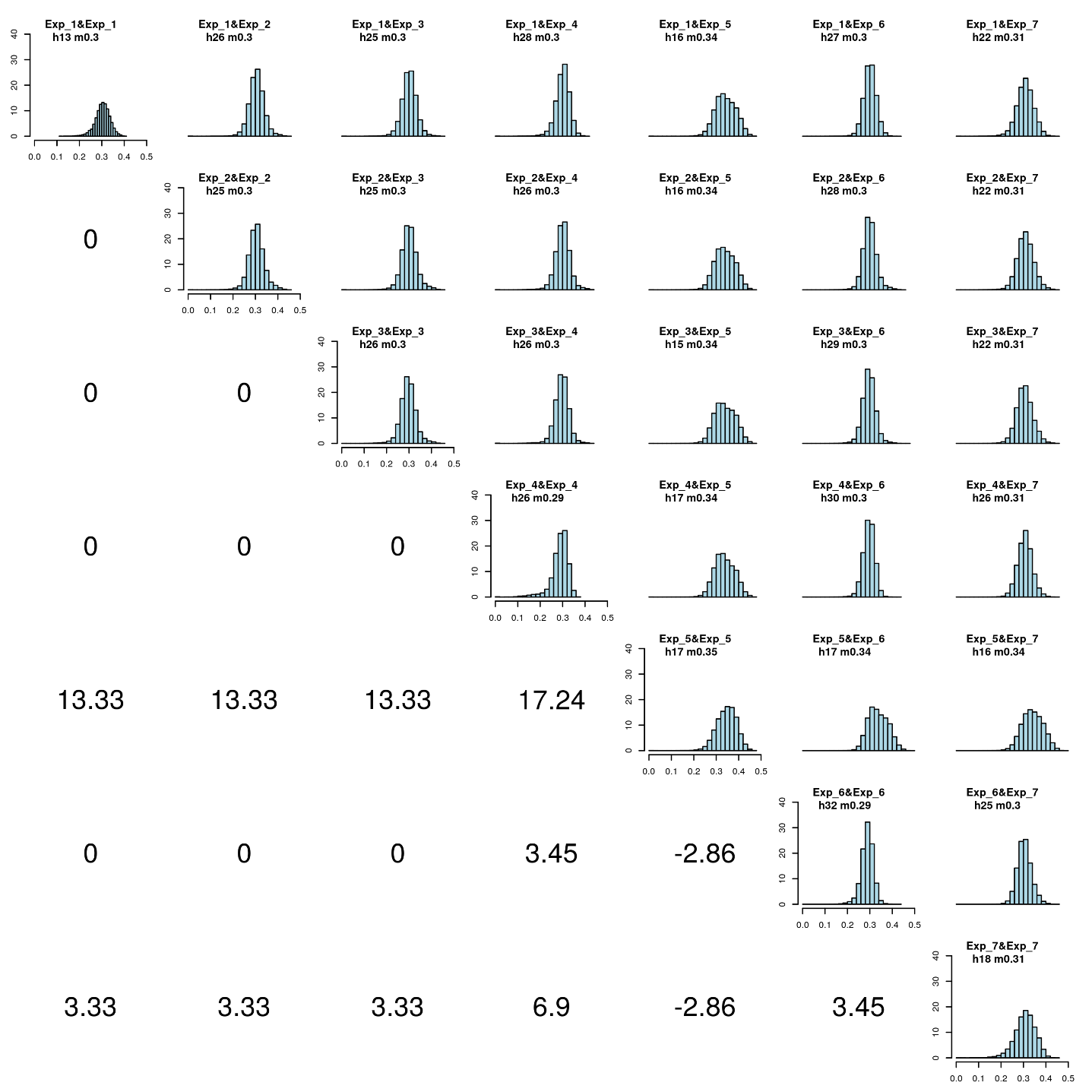


Fig. S5: Distribution of Rogers’ distances for lines within and across experimental series. For each histogram the maximum density (y-axis) and distribution mean is reported with “hxx mx.xx” below subFig S title. The off-diagonal values are the percentage changes in mean Rogers’ distance values. Higher values reflect larger genetic distance between the respective series.


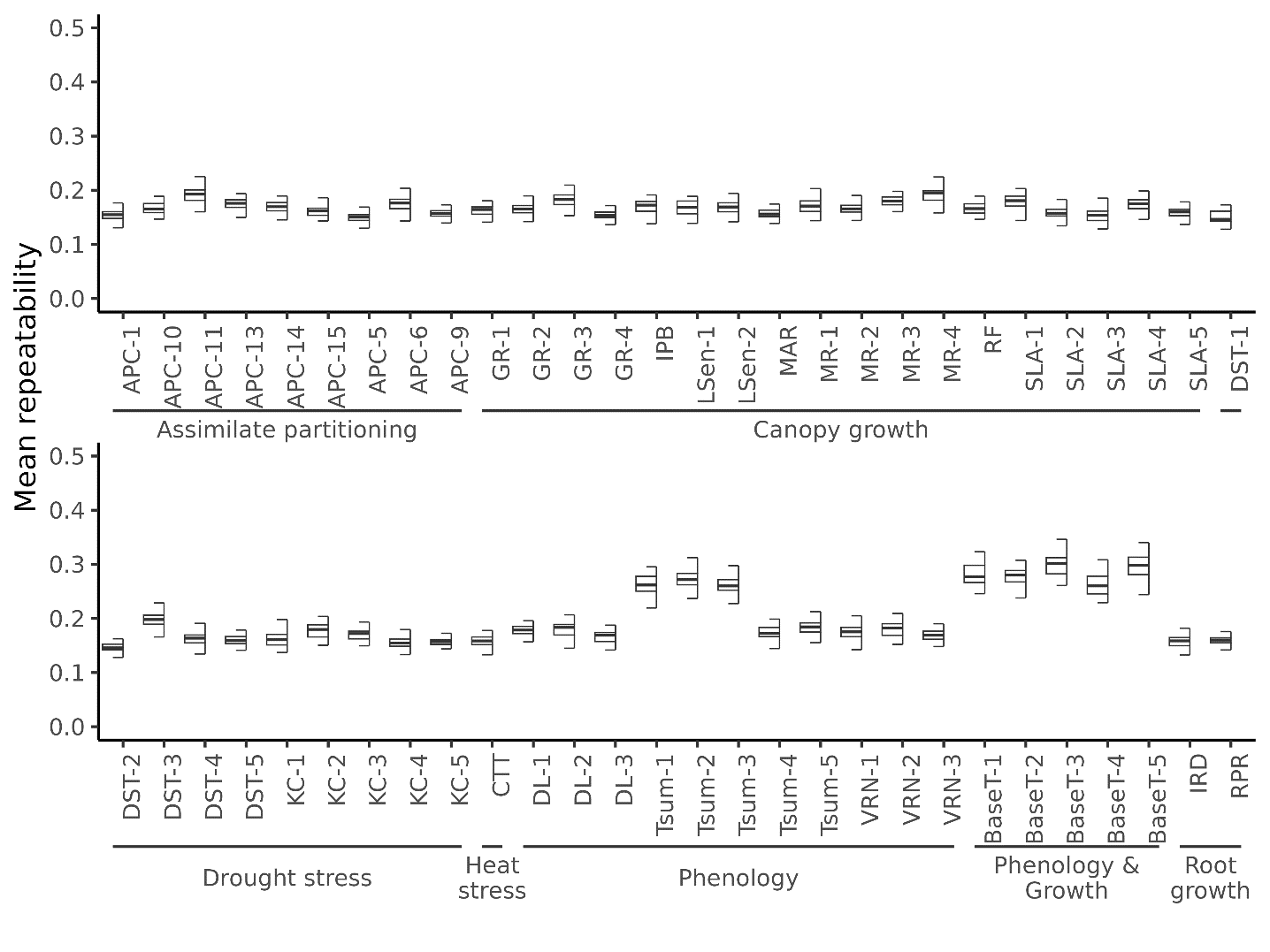


Fig. S6: Mean genomic prediction repeatabilities of MONICA parameters grouped by associated processes (see Supplementary table 7 for more information).


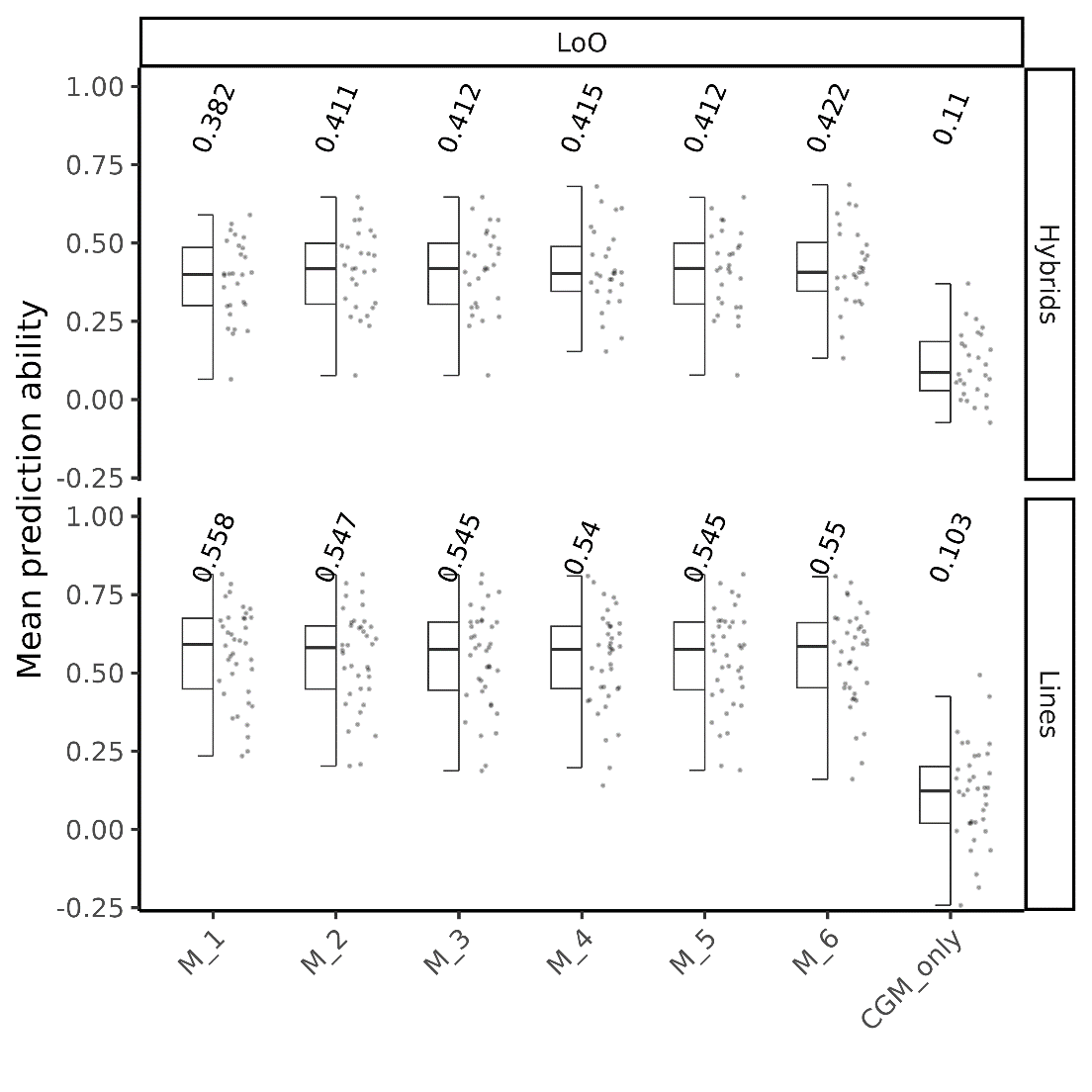


Fig. S7: Mean prediction ability of grain yield for the leave one environment out (LoO) cross validation scenario.


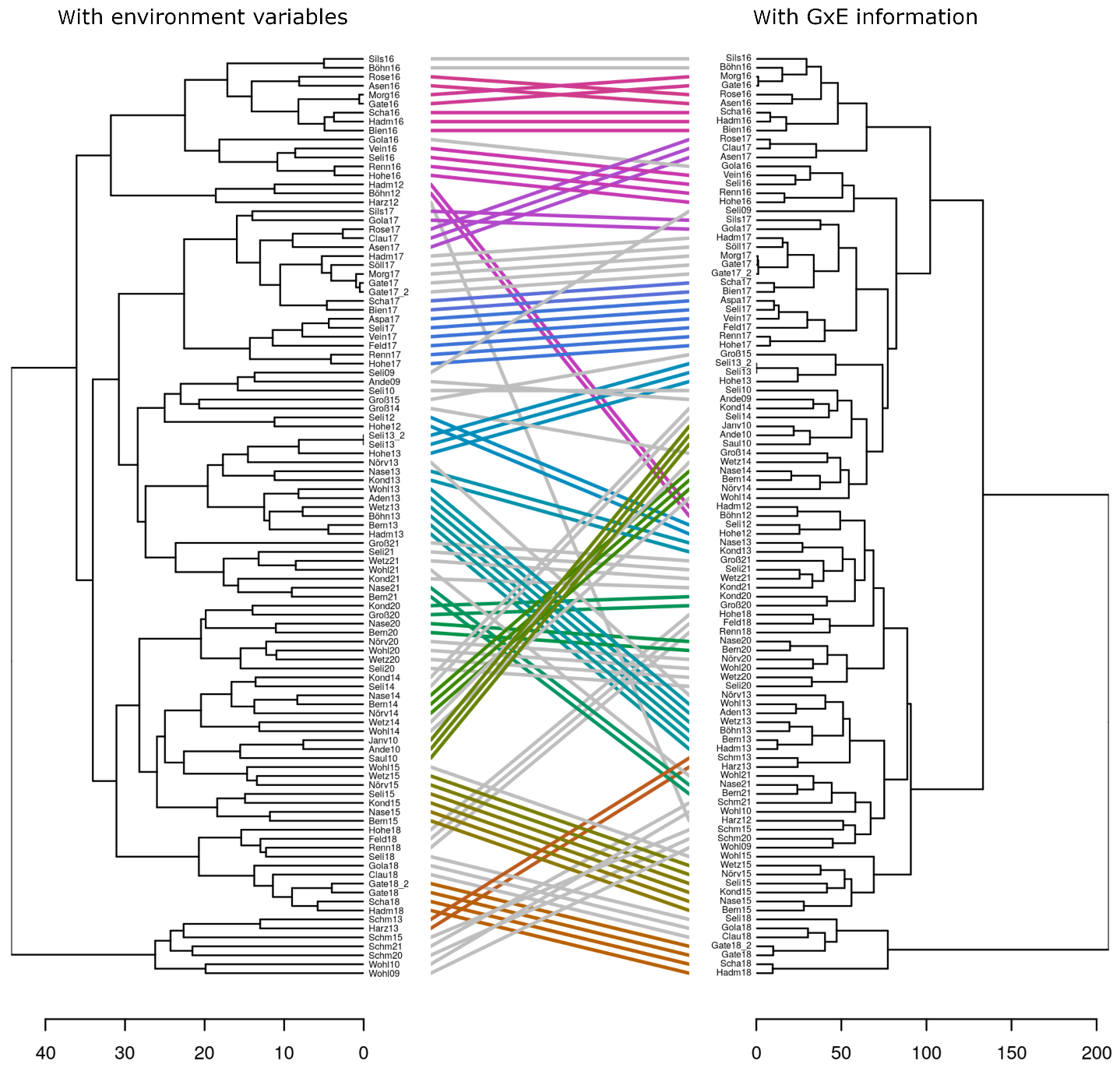


Fig. S8: Tanglegram comparing clustering of environments based (left) solely on climate data and (right) G×E data derived from model residuals. Colored lines indicate sub-trees which are present in both dendrograms. X-axis represents the distances used for splitting/merging trees for respective data types.


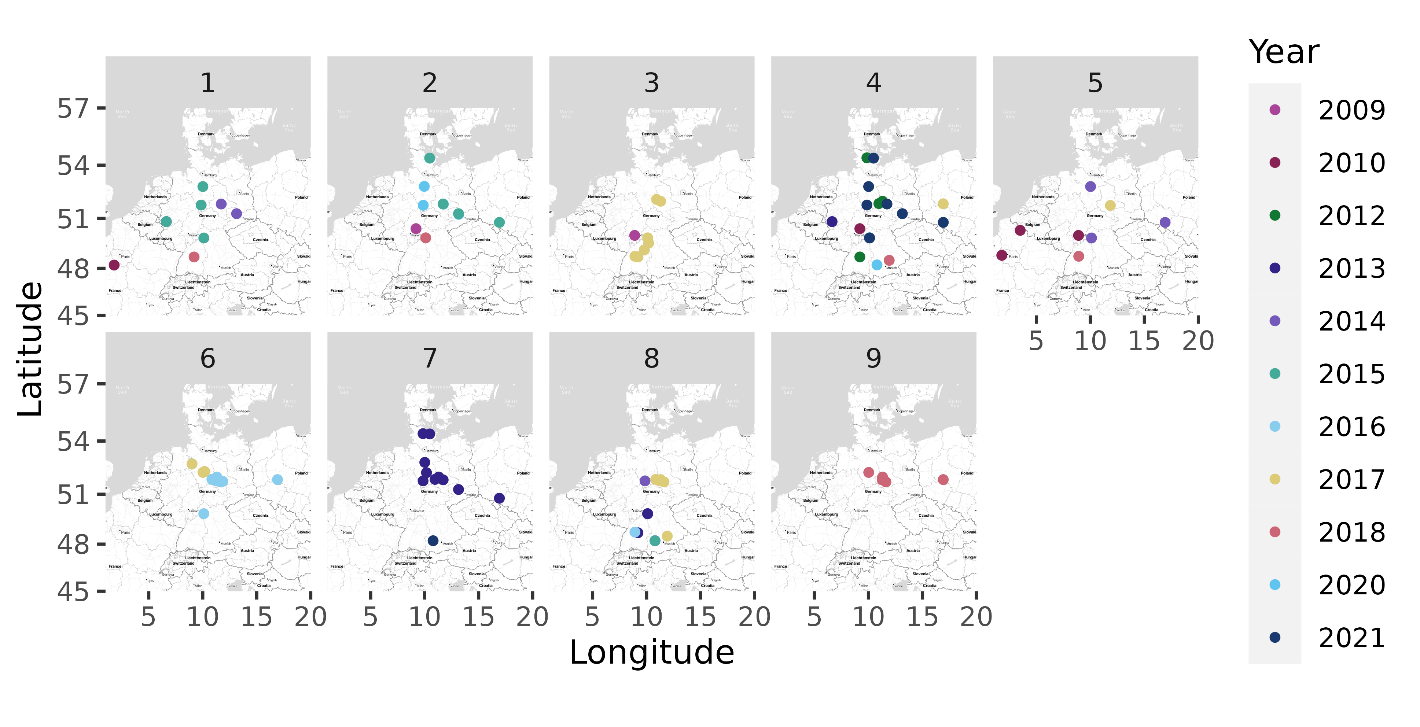


Fig. S9: Identified environment clusters projected on map of Europe. Points are the environments identified in clusters 1 to 9.


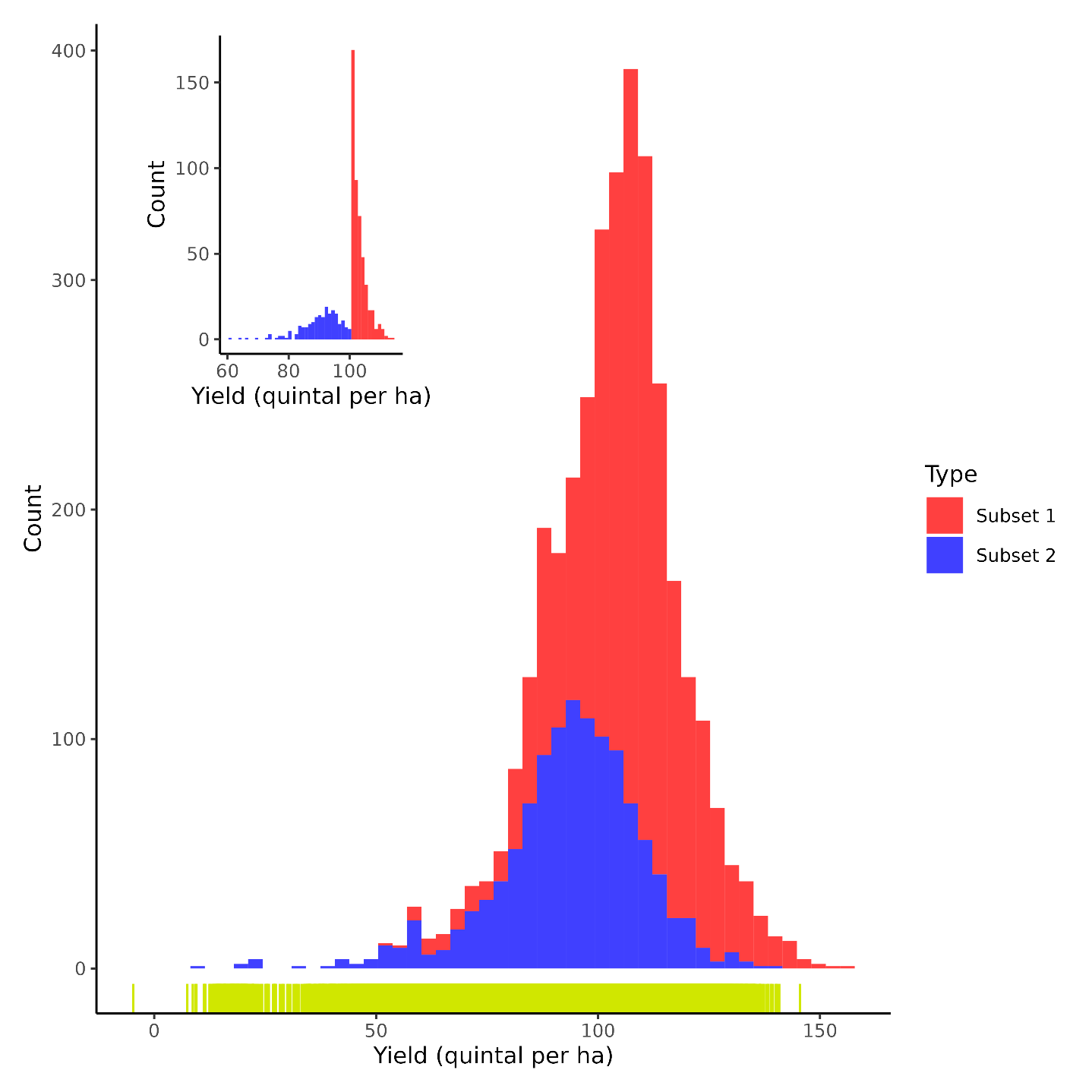


Fig. S10: Grain yield distribution of subset 1 and subset 2 selected based on average performance. Inset: average performance, Main: performance within environments, Rug plot: performance of unselected lines within environments.
