# Supplementary material for: Predicting enviromically adapted varieties with big data
Source: Genome Biol. 2026 Jan 7;27:3. doi: 10.1186/s13059-025-03914-x (PMC12838137; doi:10.1186/s13059-025-03914-x)
Supplement: Supplementary file 3 — Additional file 3. Contains additional Materials S1-9. [file 13059_2025_3914_MOESM3_ESM.docx]

Material S1: The acr_CNN model processes input through a sequential layer structure where the first convolutional and average pooling layer receives a 1-D array of scaled additive marker effects. This is followed by variable convolutional and average pooling layers, then a flattening layer and variable dense layers with dropout, leading to the output layer that makes the final prediction (Figure 1).


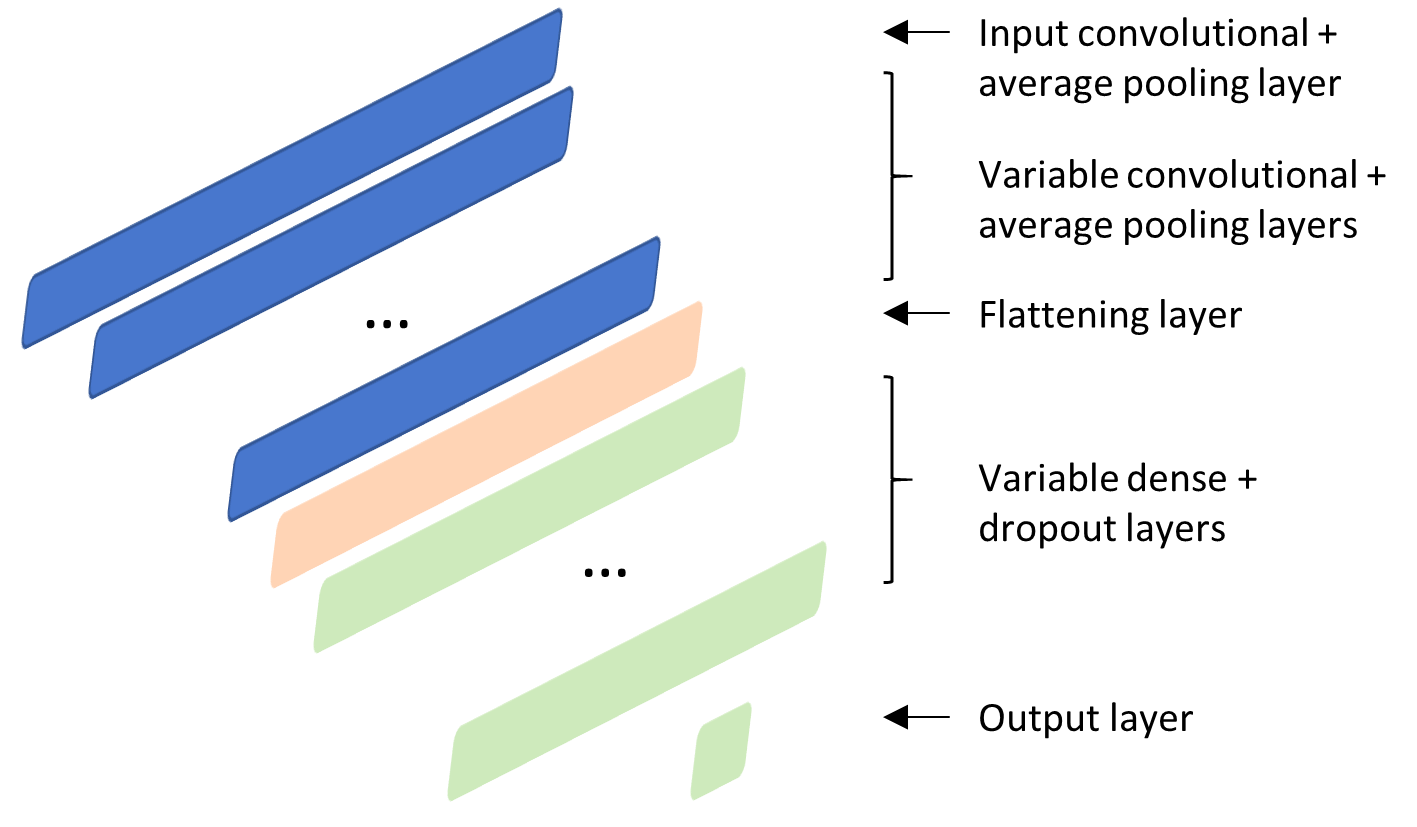


Figure 1: Architecture for acr_CNN tuner model. The architecture is illustrated using distinct colors to represent different layer types: blue indicates the input convolutional and average pooling layer as well as the variable convolutional and average pooling layers, orange represents the flattening layer, green shows the variable dense layers with dropout, and the final output layer.

The model uses ‘relu’ activation functions for convolutional and dense layers and employs the “Adam” optimizer for training [1]. The number of variable layers and all hyperparameters were determined through hyperparameter tuning, with specific parameter ranges and defaults detailed in Table 1.

Table 1: Hyperparameter space for acr_CNN

| Component | Hyperparameter | Range | Default | Step |
| --- | --- | --- | --- | --- |
| Input convolutional +  average pooling layer | Number of filters | 64-512 | 512 | 64 |
|  | Kernel size | 3-36 |  | 3 |
|  | Average pooling size | 2-32 | 16 | 4 |
| Variable convolutional +  avearge pooling layers | Number of layers | 2-4 |  | 1 |
|  | Number of filters | 64-512 | 256 | 32 |
|  | Kernel size | 3-36 |  | 3 |
|  | Average pooling size | 2-32 | 16 | 4 |
| Dense Layers | Number of layers | 1-4 |  | 1 |
|  | Number of units | 32-256 | 128 | 32 |
|  | Dropout rate | 0.1-0.5 |  | 0.01 |
| Optimizer | Learning rate | 10^-5^ to 10^-2^ |  |  |
|  | Beta 1 | 0-1 |  |  |
|  | Beta 2 | 0-1 |  |  |

Material S2: Heading date data was added for 92 out of 117 environments. The protocol for phenotyping HD varied between data originating from 2012-2015 in Exp_6 and the rest of the series (from Exp_6 and other series). The former was recorded as BBCH stage [2] on the day when the ears of approximately half of the plants in a plot were fully visible, while in the rest of the series it was recorded as days from 1st January of the harvest year. The BBCH data were converted to days from 1st of January of the harvest year in order to harmonize the HD data. For this, mean values were calculated for 40 common checks in the data where the heading dates were recorded as BBCH stage as well as days from 1 January. A linear model was then fit, regressing the former on the latter. With the trained model, the days from 1 January were predicted for all genotypes within each environment for Exp-6 data originating from years 2012 to 2015. The model fit had an adjusted R^2^ value of 0.46, with the assumption that the relation between BBCH records and heading date in days from 1 January of harvest year is linear.

To fill the partial missing HD values for 51 out of 92 environments (mean missing proportion = 4.24 %), a genomics-based prediction approach was employed. Here, an GBLUP_D model was fit that regressed heading dates on additive and dominance effect matrices derived from integrated genomic data, using the BGLR package [3] with a burn-in of 2,000 iterations and a total of 12,000 iterations. For a given environment, genotypes with non-missing heading dates were set as training set and the values in the remaining i.e. test set were predicted. The prediction accuracies were assessed by calculating the correlation between the observed and predicted HD values of the training set. The training set prediction accuracy for prediction of heading dates in environments with partial data was high (median = 0.87, min = 0.72, max = 0.98 for a given environment) overall, indicating good model fit. Lastly, we checked data quality for all environments with genomic repeatabilities i.e. ratio of genomic variance to the sum of genomic and error variances, estimated using GBLUP model [4] (Table 2).

Table 2: Genomic repeatabilities for heading date.

| Series | Trait | Min | Median | Mean | Max | Environments |
| --- | --- | --- | --- | --- | --- | --- |
| Exp_1 | heading date | 0.558 | 0.714 | 0.690 | 0.747 | 8 |
| Exp_2 | heading date | 0.600 | 0.762 | 0.752 | 0.869 | 10 |
| Exp_3 | heading date | 0.691 | 0.752 | 0.748 | 0.807 | 5 |
| Exp_4 | heading date | 0.332 | 0.585 | 0.574 | 0.750 | 22 |
| Exp_5 | heading date | 0.160 | 0.560 | 0.517 | 0.666 | 11 |
| Exp_6 | heading date | 0.226 | 0.416 | 0.416 | 0.653 | 28 |
| Exp_7 | heading date | 0.556 | 0.602 | 0.607 | 0.666 | 8 |

Material S3: Proportion of phenotypic variance captured by respective BLUP based model(s) revealed a clear trend, with reducing error variance component from M_1 to M_6 (Table 3). Aggregating $G_{a}$, $G_{d}$, $G_{aa}$, it is evident that M_2, M_3 and M_5 captured approximately 66% percent of the baseline genetic variance [e.g. (6.28 + 1.9 + 3.89)/18.26 for M_2]. M_4 and M_6 on the other hand capture approximately 74.3% of the baseline genetic variance [e.g. (5.3 + 1.54 + 2.43 + 4.37)/18.3]. Notably, around 32% of the latter is attributed due to G×E effects alone.

Table 3: Component model variances.

| Component | M_1 | M_2 | M_3 | M_4 | M_5 | M_6 | M_7 | M_8 |
| --- | --- | --- | --- | --- | --- | --- | --- | --- |
| $E_{I}$ | 63.95 | 60.51 |  |  |  |  |  |  |
| $G_{I}$ | 18.26 |  |  |  |  |  |  |  |
| $G_{a}$ |  | 6.28 | 6.29 | 5.3 | 6.3 | 2.61 | 6.61 | 3.71 |
| $G_{d}$ |  | 1.9 | 1.91 | 1.54 | 1.91 | 1.52 | 1.39 | 1.42 |
| $G_{aa}$ |  | 3.89 | 3.89 | 2.43 | 3.9 | 2.16 |  |  |
| $E_{l}$ |  |  | 258.13 | 243.08 |  |  |  |  |
| $E_{nl}$ |  |  |  |  | 207.2 | 198.1 |  |  |
| $G_{a}$⊙$E_{l}$ |  |  |  | 4.37 |  |  | 5.07 | 4.81 |
| $G_{a}$⊙$E_{nl}$ |  |  |  |  |  | 7.21 |  |  |
| $Y$ |  |  |  |  |  |  | 1604.91 | 222.12 |
| $S$ |  |  |  |  |  |  | 394.26 | 199.06 |
| $G_{a}$⊙$Y$ |  |  |  |  |  |  | 2.3 | 1.27 |
| $GS_{I}$ |  |  |  |  |  |  | 6.43 |  |
| $GS$ |  |  |  |  |  |  |  | 0.61 |
| err_var | 28.44 | 24.5 | 24.56 | 24.18 | 24.51 | 24.11 | 21.17 | 16.84 |

Material S4: The CNN_EV model processes input through three connected modules in an acyclic directed graph structure where the genomic convolution module processes scaled additive marker effects through three 1-D convolutional and average pooling layers, while the environment variable convolution module processes environmental data through three 1-D convolutional layers. The outputs from both modules are sequentially appended and concatenated, then fed to the deep layer module containing fixed input dense layers with dropout, variable dense layers with dropout, and a fixed output layer that produces the final prediction (Figure 2).

The model uses ReLU activation functions for convolutional and dense layers and employs the Adam optimizer for training, with hyperparameters for the deep layer module determined through hyperparameter tuning, with specific parameter ranges and defaults detailed in Table 4 and 5.


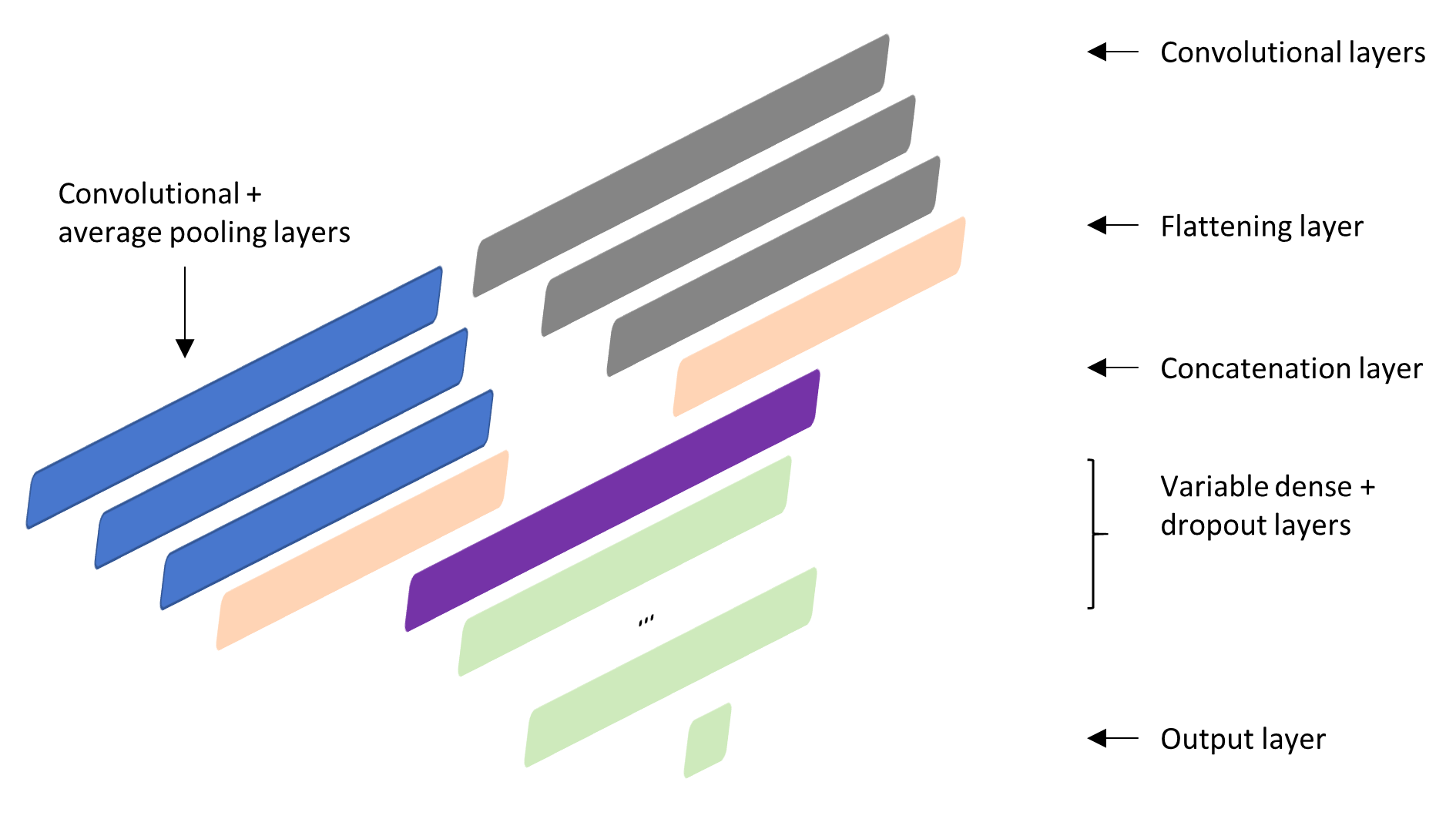


Figure 2: Architecture for CNN_EV tuner model. The architecture is illustrated using distinct colors to represent different layer types and modules: blue indicates the convolutional and average pooling layers in the genomic convolution module, gray represents the convolutional layers in the environment variable convolution module, orange shows the flattening layer, purple depicts the concatenation layer where outputs from both modules are combined, green illustrates the variable dense layers with dropout in the deep layer module, and the final output layer.

Table 4: Hyperparameter space for CNN_EV/CNN_GS: Convolutional module

| Component | Number of filters | Kernel size | Average pooling size | Average pooling Stride |
| --- | --- | --- | --- | --- |
| E*: Layer 1 | 128 | 2 |  |  |
| E*: Layer 2 | 64 | 2 |  |  |
| E*: Layer 3 | 32 | 2 |  |  |
| G**: Layer 1 | 512 | 8 | 8 | 4 |
| G**: Layer 2 | 256 | 6 | 6 | 3 |
| G**: Layer 3 | 128 | 4 | 4 | 2 |

*Environment variable, G**Marker effects

Table 5: Hyperparameter space for CNN_EV/CNN_GS: Densely connected module

| Component | Hyperparameter | Range | Default | Step |
| --- | --- | --- | --- | --- |
| Dense Layers | Number of layers | 1-5 |  |  |
|  | Number of units | 64-1024 | Varied per layer | Varied per layer |
|  | Dropout rate | 0.1-0.5 | 0.2 | 0.01 |
| Optimizer | Learning rate | 10^-5^ to 10^-2^ |  |  |
|  | Beta 1 | 0-1 |  |  |
|  | Beta 2 | 0-1 |  |  |

Material S5: Across the prediction scenarios, RMSE values showed an increasing trend, reflecting greater modeling complexity from scenarios cv1 to cv3, cv2 and cv4 (Figure 3). The lowest overall RMSE values were observed in cv1, with values of 4.0 for Hybrids and 5.8 for Lines, followed by cv3 (4.5 and 6.6), cv2 (11.6 and 11.3), and cv4 (11.5 and 11.6), respectively. This indicates that predictive performance decreased as the complexity of the prediction scenario increased, with cv4 representing the most challenging setting. Within cv1, model M_8 achieved the lowest mean RMSE value, closely followed by M_6. Model M_6 also performed best in cv3. In cv2 and cv4, a broader spread of RMSE values likely contributed to higher variability and less reliable mean estimates. Interestingly, however, the values recorded with CNN_EV were among the among the lowest RMSE in these scenarios.


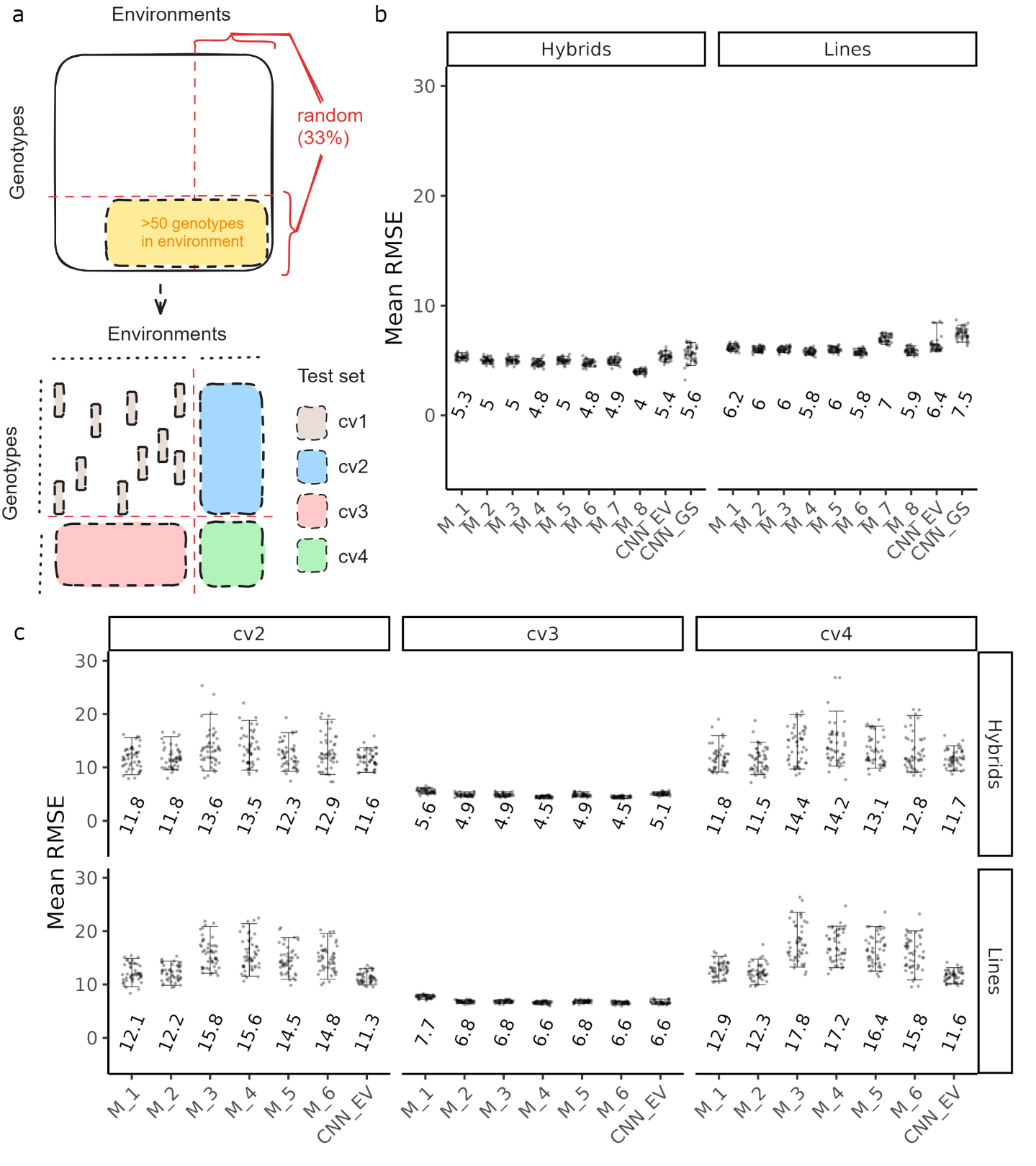


Figure 3: Overview for enviromically adapted genomic predictions. Schematic of (a) cross-validation scheme: As a first step environments with more than 50 genotypes were filtered. The remaining data were then split into four quadrants (shown by intersecting red lines) with fourth quadrant (bottom right) containing 33 percent of unique dataset genotypes in 33 percent of unique dataset environments. A given run comprised cv1 to cv4. Test set for cv1 was a 20 percent random sample from quadrant 1, while complete quadrant 2,3 or 4 was used as test sets for cv2, cv3 and cv4, respectively. The training set for a run was the remaining 80 percent sample from quadrant 1. Mean root mean square value (RMSE) for grain yield with cross validation scenarios (b) cv1 and (c) cv2, cv3 and cv4. RMSE was calculated for each test set environment, cross validation scenario, and run. Thereafter, mean RMSE value was reported for each cross validation scenario and run. Results are shown separately for hybrids and lines. Error bars represent 5^th^ to 95^th^ percentile range.

Material S6: The plot-based values from respective environments were corrected for statistical experimental design effects following,

$$y_{ijkl}= \mu+ g_{i}+t_{j}+r_{jk}+b_{jkl}+e_{ijkl}$$

where $y$ is the plot-based grain yield data, $\mu$ is the overall mean, $g_{i}$ is the genotype effect, $t_{j}$, $r_{jk}$, and $b_{jkl}$ are design effects for trial, replication and block, respectively. $e_{ijkl}$ is the residual effect and *i*, *j*, *k* and *l* are indices for the corresponding model effects. Depending on the experimental design at an environment, only some or none of the $t_{j}$, $r_{jk}$, and $b_{jkl}$ effects were estimated. When the experimental design was unreplicated, respective design effects were estimated and subtracted from $y_{ijkl}$. Trial effects were always modelled as random effect, whereas $g_{i}$ was modelled as random when estimating genotype variances. To derive within environment best linear unbiased estimates, $g_{i}$ was modelled as a fixed effect. All random effects were assumed to be normally, identically and independently distributed. Outlier correction was performed on a plot basis with “Bonferroni-Holm with rescaled median absolute deviation standardized residuals” [5]. The repeatability values were calculated for environments where replicated data were available.

Material S7: A two-step experimental design correction was done to obtain design effect corrected phenotypic data for advanced winter wheat lines in late selection stages from KWS Lochow GmbH. The trials at each site were conducted on advanced winter wheat lines, selected from three stages of a breeding program: three years before registration, two years before registration, and one year before registration. Briefly, each trail at a given site was connected to others with at least 10 checks. The BLUEs per environment were derived in a two-step process where (1) design and trial effects were corrected for a given selection stage, (2) effect of selection stage was corrected for sites where phenotypic data of multiple selection stages was available. Outlier correction was performed on a plot basis for the first step with “Bonferroni-Holm with rescaled median absolute deviation standardized residuals” [5]. The repeatability values were calculated for environments where replicated data was available.

Material S8: Out of 81,587 possible markers from the reference chip, 67,065 were mapped successfully to the reference genome and their reference as well as alternative state information was therefore available. SNP array data were first harmonized to HapMap format [6] but without SNP information typically identified with first eleven columns of HapMap format. Instead, genotype identifiers in one row per genotype and SNP identifiers in one column per marker were used to identify a unique SNP array data point. SNP and genotype identifiers overlapping with the reference array (chip 9) from all chips excepting the reference array were then identified. Six out of the nine arrays, excluding the reference array, were derived from KWS SAAT SE & Co. KGaA and had marker calls inverted [ATGC] to [TACG] for a subset of markers across the six arrays. These two sets of arrays (company and non_company) were therefore compared for their respective SNP calls to assert a unified call at a given marker. For the latter, common genotypes between the two sets of arrays were assessed and if calls were exactly inverted in company arrays compared to non_company arrays, the SNP calls in company arrays were reverted. After correction of invert status, arrays were merged first marker-wise and then across markers to get a unified marker array. Naturally, markers where marker positions were not available were omitted. An SNP genotype dataset was then created from this combined matrix with snpgdsCreateGeno [7], the dataset was converted to a .gds format using seqSNP2GDS [8], and finally to a .vcf using seqGDS2VCF [8] function(s). Out of the 67,065 markers in the raw vcf, 1,891 markers were removed due to completely missing genotype data. Filtering for more than 50 percent missing values further removed 54,873 markers, leaving 10,301 markers for imputations.

Material S9:

${GRM}_{a} =\frac{WW^{T}}{2\sum_{k = 1}^{p} p_{k}(1-p_{k})}$,

${GRM}_{aa}$= ${GRM}_{a}\odot{GRM}_{a}$

If $X= (x_{ij})$ is a $n \times p$ matrix derived from integrated marker data, where $x_{ij}$ represents the number of reference alleles for the $i$th genotype at the $j$^th^ marker, then $W= (x_{ij} - 2p_{j})$, $W^{T}$ denotes the transpose of $W$, and $p_{j}$ is the reference allele frequency at the $j$th marker. The symbol “$\odot$” denotes a Hadamard product to approximate first degree epistasis interaction effects. We first define $V$ as the general orthogonal design matrix for dominance marker effects [9], if $f_{11}$, $f_{12}$, $f_{22}$ are the genotype frequencies at $j$^th^ marker, then $V=(V_{ij})$ can be derived from $X$ by substituting {0,1,2} to {$v_{1}$,$v_{2}$,$v_{3}$}, where

$$v_{1}= -\frac{2f_{12}f_{22}}{f_{11}+f_{22}-{(f_{11}-f_{22})}^{2}}$$

$$v_{2}= \frac{4f_{11}f_{22}}{f_{11}+f_{22}-{(f_{11}-f_{22})}^{2}}$$

$$v_{3}= -\frac{2f_{11}f_{12}}{f_{11}+f_{22}-{(f_{11}-f_{22})}^{2}}$$

Then,

$${GRM}_{d} =\frac{VV^{T}}{mean(diagonal(VV^{T}))}$$

Where, $V^{T}$ denotes the transpose of $V$.

# References

1. Martín Abadi, Ashish Agarwal, Paul Barham, Eugene Brevdo, Zhifeng Chen, Craig Citro, et al. TensorFlow: Large-Scale Machine Learning on Heterogeneous Systems [Internet]. 2015. Available from: https://www.tensorflow.org/

2. Zadoks JC, Chang TT, Konzak CF. A decimal code for the growth stages of cereals. Weed Research. 1974;14:415–21.

3. Pérez P, de Los Campos G. Genome-wide regression and prediction with the BGLR statistical package. Genetics. 2014;198:483–95.

4. Jiang Y, Reif JC. Modeling epistasis in genomic selection. Genetics. 2015;201:759–68.

5. Bernal-Vasquez A-M, Utz H-F, Piepho H-P. Outlier detection methods for generalized lattices: a case study on the transition from ANOVA to REML. Theoretical and Applied Genetics. 2016;129:787–804.

6. Gibbs RA, Belmont JW, Hardenbol P, Willis TD, Yu F, Yang H, et al. The International HapMap Project. Nature. 2003;426:789–96.

7. Zheng X, Levine D, Shen J, Gogarten S, Laurie C, Weir B. A High-performance Computing Toolset for Relatedness and Principal Component Analysis of SNP Data. Bioinformatics. 2012;28:3326–8.

8. Zheng X, Gogarten S, Lawrence M, Stilp A, Conomos M, Weir B, et al. SeqArray – A storage-efficient high-performance data format for WGS variant calls. Bioinformatics. 2017;

9. Alvarez-Castro JM, Carlborg O. A unified model for functional and statistical epistasis and its application in quantitative trait loci analysis. Genetics. 2007;176:1151–67.
